# Constructing Bayesian networks by integrating gene expression and copy number data identifies *NLGN4Y* as a novel regulator of prostate cancer progression

**DOI:** 10.18632/oncotarget.11925

**Published:** 2016-09-10

**Authors:** Yixuan Gong, Li Wang, Uma Chippada-Venkata, Xudong Dai, William K. Oh, Jun Zhu

**Affiliations:** ^1^ The Tisch Cancer Institute, Division of Hematology and Medical Oncology, Icahn School of Medicine at Mount Sinai, New York, NY, USA; ^2^ Icahn Institute for Genomics and Multiscale Biology, Icahn School of Medicine at Mount Sinai, New York, NY, USA; ^3^ Department of Genetics and Genomic Sciences, Icahn School of Medicine at Mount Sinai, New York, NY, USA

**Keywords:** Bayesian networks, NLGN4Y, prostate cancer, negative regulator, gene expression

## Abstract

To understand the heterogeneity of prostate cancer (PCa) and identify novel underlying drivers, we constructed integrative molecular Bayesian networks (IMBNs) for PCa by integrating gene expression and copy number alteration data from published datasets. After demonstrating such IMBNs with superior network accuracy, we identified multiple sub-networks within IMBNs related to biochemical recurrence (BCR) of PCa and inferred the corresponding key drivers. The key drivers regulated a set of common effectors including genes preferentially expressed in neuronal cells. NLGN4Y—a protein involved in synaptic adhesion in neurons—was ranked as the top gene closely linked to key drivers of myogenesis subnetworks. Lower expression of *NLGN4Y* was associated with higher grade PCa and an increased risk of BCR. We show that restoration of the protein expression of NLGN4Y in PC-3 cells leads to decreased cell proliferation, migration and inflammatory cytokine expression. Our results suggest that *NLGN4Y* is an important negative regulator in prostate cancer progression. More importantly, it highlights the value of IMBNs in generating biologically and clinically relevant hypotheses about prostate cancer that can be validated by independent studies.

## INTRODUCTION

Prostate cancer (PCa) is the most frequently diagnosed cancer in American men [1]. However, it is a very heterogeneous disease, with a phenotype ranging from indolent behavior lasting decades to highly aggressive metastatic cancer which can be lethal in just a few years. Although most patients with advanced disease initially respond to surgical or chemical depletion of serum testosterone, PCa invariably progresses, a clinical state known as castration-resistant prostate cancer (CRPC). With the emergence of new treatment options, survival of patients with CRPC has significantly improved in the past few years. However, CRPC remains highly lethal, and a thorough understanding of the genetic drivers of PCa progression will help in the clinical management of the disease.

Some of the key challenges of PCa research include how to identify patients at high risk for early progression and how to prevent it. The key pathways leading to prostate cancer progression are not fully understood. Large scale genomic studies have been conducted to uncover novel genetic drivers of aggressive PCa [2] through analyzing gene expression datasets [3–7], identifying copy number alterations (CNAs) [8–10] and gene fusions [11–13], and detecting somatic mutations [14]. However, most of these studies focused on only one type of data, and when multiple data types were profiled, analysis was generally conducted for individual genes separately or within known pathways [15]. Since multiple genes and pathways are involved in cancer progression, systems level analysis is needed to understand how such genes interact with and/or regulate each other, and also how multiple genes and pathways work together to determine clinically meaningful endpoints such as disease recurrence.

**Figure 1:**
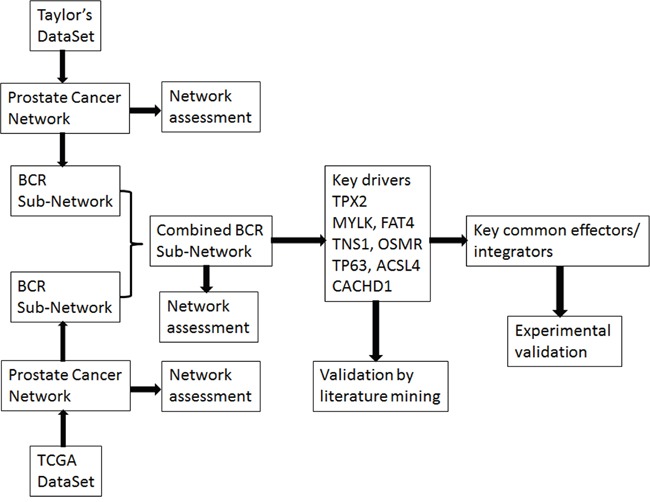
The workflow of the study

We previously developed an analytical procedure, RIMBANET [16], to construct integrative molecular Bayesian networks (IMBNs) by integrating genetic and genomic profiling data under the framework of a Bayesian network. This integrative approach has been successfully used in dissecting causal relationships in complex human diseases such as breast cancer [17], hepatocellular carcinoma [18], diabetes and obesity [19], as well as in other diseases [16]. Integration of diverse types of data with gene expression data can improve network accuracy [16] with the directed network representing *biologically meaningful* causal relationships [20] as opposed to sheer statistical relationships. In this study (the workflow shown in Figure 1), we developed a similar approach to integrating gene expression and CNA data and applied it to two of the largest comprehensive genomic datasets available for PCa. We leveraged the constructed IMBNs for PCa to identify novel genes and pathways underlying PCa recurrence.

## RESULTS

### Construction of IMBNs from two independent PCa datasets

We reconstructed IMBNs for PCa based on two of the largest published PCa datasets—the Taylor dataset (150 samples) [15] and the TCGA PRAD dataset (432 samples) [21]. The two datasets differed significantly in terms of patient characteristics (Table 1), and detailed description can be found in Supplementary Methods. For example, more than half (53.7%) of patients in Taylor's dataset have a Gleason score <=6, while the fraction is only 8.6% for the TCGA dataset. On the other hand, 26.3% of patients in the TCGA dataset have a Gleason score >=9; the fraction is only 6% for Taylor's dataset. The median follow-up time for Taylor's dataset is much longer than the TCGA dataset. As a result, the percentage of patients with BCR (25.7%) is much higher for TCGA (15.4%), even though most of patients in Taylor's dataset are in better prognosis groups (as defined by Gleason scores). The platforms used to generate the datasets were also different (Table 1). mRNA expression was profiled using Affymetrix Exon array in the Taylor dataset, and Illumina HiSeq for RNA-seq in the TCGA dataset, respectively. The CNA was profiled using Agilent CGH array in the Taylor dataset, and Affymetrix SNP array for the TCGA dataset, respectively.

**Table 1:** Characteristics of the two prostate cancer datasets and the corresponding networks

		Taylor	TCGA
Sample Size		150^&^	498*
Gleason Score <=6		80 (53.7%)	37 (8.5%)
Gleason Score =7		50 (33.6%)	224 (51.3%)
Gleason Score =8		10 (6.7%)	58 (13.3%)
Gleason Score >=9		9 (6.0%)	118 (27.0%)
Median follow-up time		45.5 months	16.2 months
Percentage of patients with BCR		25.7%	15.4%
Gene expression platform		Affymetrix Human Exon 1.0 ST array	Illumine HiSeq RNAseq
CN platform		Agilent 244K comparative genomic hybridization array	Genome wide SNP Array 6
IMBNs	#Genes included	6,798	8,896
	#Cis genes	153	3,003
	#Gene-gene regulations	8,064	14,418

& Number of patients with gene expression data available in Taylor's dataset. Gleason score was not available for 1 of them, and BCR data was not available for 10 of them.

* Number of patients with gene expression data available in TCGA dataset. Gleason score was not available for 61 of them, and BCR data was not available for 114 of them.

Due to the obvious difference of the two datasets, we didn't combine them in the network reconstruction process. Instead, we reconstructed IMBNs from each of the two datasets separately by integrating its gene expression and CNA data. The basic characteristics of the two reconstructed IMBNs are listed in Table 1. 6,798 and 8,896 informative genes (Supplemental Methods) were included in reconstructing IMBNs for the Taylor and the TCGA datasets, respectively. Among the informative genes, 3609 genes were common (Fisher's exact test *p* = 1 × 10^−52^). More cis-CNAs (Supplemental Methods) were identified in the TCGA dataset compared to Taylor's dataset (Table 1). Among 157 cis-CNAs identified in the Taylor's dataset, 127 were identified in both datasets (Fisher's exact test *p* = 1.2 × 10^−51^), suggesting that the difference of numbers of cis-CNAs identified in the two datasets is due to a higher statistical power of the TCGA dataset as there were more samples in the TCGA dataset.

### Comparison of IMBNs reconstructed from the two PCa datasets

Although the two PCa datasets differ considerably in multiple aspects, the IMBNs reconstructed from the two datasets share significant similarities. First, the degrees of each gene (defined as the number of close neighbors; see Supplemental Methods for details) in the two networks are significantly correlated (Spearman's correlation r= 0.28, *p* = 8.5 × 10^−69^). Second, for the majority (59.9%) of genes common in the two IMBNs, their network neighbors significantly overlap (Fisher's exact test p<0.05) with each other in the two networks (Noted in Supplemental Methods). The fraction is even higher for genes with higher degrees (Supplementary Figure S1). For the top 20% genes ranked by node degree, 81% share significantly overlapping network neighbors between the two IMBNs.

### Advantage of integrating CNA data in reconstructing IMBNs

To assess the accuracy of reconstructed networks, we compared our IMBNs with several widely used databases of gene networks and gene sets (Supplemental Methods). Specifically, we calculated the percentage of our inferred gene-gene regulations that are in existing protein/gene network databases, or within the same pathway in gene set databases. For all the reference databases considered, the estimated accuracy of our IMBNs is significantly better than random networks (by permuting gene names in the IMBNs) (Supplementary Table S1). Furthermore, the accuracy of the TCGA PRAD IMBN reconstructed by integrating gene expression and CNA data is consistently higher than that of the IMBN reconstructed from gene expression data only (Supplementary Table S1). However, the contribution of CNA data is inconsistent for the Taylor dataset (Supplementary Table S1) due to a small number of cis-CNAs included in reconstructing the IMBN (Table 1). Interactions in the STRING database [22] are biased to coexpressed gene pairs. One of our goals in integrating CNA data is to distinguish gene-gene coexpression due to co-localization in the same CNA blocks or due to transcriptional regulation [23]. On the other hand, interactions in the HumanNet database [24] are selected taking over 20 different types of “-omics” data into consideration. The Taylor IMBN with CNAs is slightly better with regard to the HumanNet database, but slightly worse with regard to the STRING database than the IMBN without CNAs, which is consistent with what was expected. It is worth noting that overlap between our IMBNs and the HPRD, HumanNet, and STRING databases, which are based on protein-protein interactions, is much lower than the overlap between our IMBNs and the KEGG [25], MSigDB [26], and GO [27] databases, which are based on biological pathways (Supplementary Table S1), suggesting that regulation inferred in our IMBNs is very different from physical interaction. In summary, these results indicate that integrating gene expression and CNA data in reconstructing cancer networks improves the accuracy of resulted networks. Thus, we used only the IMBNs with CNAs integration in our further analyses.

### Evaluation of IMBNs using known PCa causal genes: ERG, AR and others

Known prostate cancer genes (Supplemental Methods) have significantly higher node degrees compared with others (Wilcoxon rank sum test p= 1.3 × 10^−5^ and 0.005 for TCGA and Taylor IMBN, respectively). We compiled a list of “high confidence” cancer genes associated with prostate cancer progression or metastasis from multiple studies (Supplementary Table S2 and S3; see Supplemental Methods for details). The node degree of these prostate cancer genes is significantly higher than that of the others (Wilcoxon rank sum test p= 9.9 × 10^−13^ and 1.1 × 10^−6^ for TCGA and Taylor IMBN, respectively). It is worth noting that the p-values above are more significant for IMBNs reconstructed by integrating gene expression and CNA data, as compared to IMBNs reconstructed from gene expression only.

**Figure 2:**
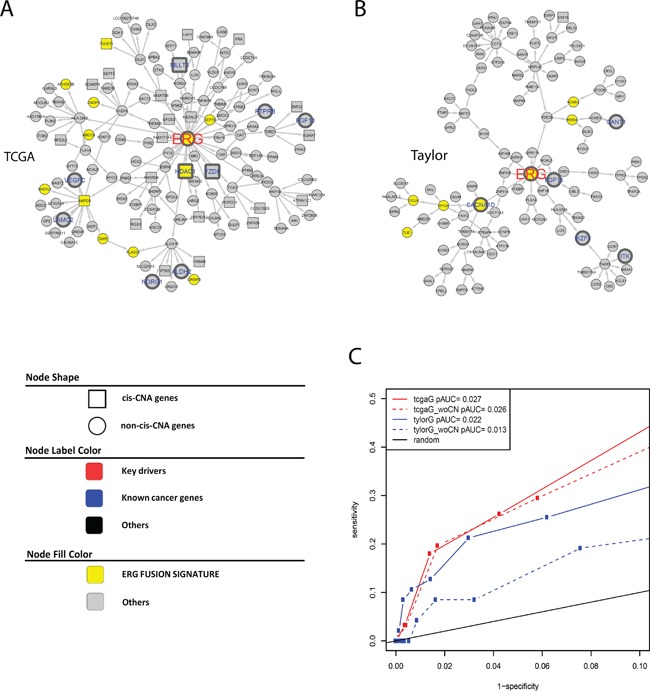
**A** and **B.** ERG subnetworks extracted from TCGA IMBN (A) and Taylor IMBN (B). Nodes of yellow color represent previously identified ERG fusion signature genes. Genes known to be cancer related are labeled in larger font size with blue color (nodes with thicker boarder). Square nodes represent genes regulated by cis CNA. **C.** The accuracy of ERG subnetworks is assessed using previously identified ERG fusion signature genes.

There are 408 and 308 key regulators in the TCGA and Taylor IMBNs, respectively (Supplemental Methods). They are enriched for prostate cancer genes (Fisher's exact test p= 5.0 × 10^−9^ and 0.002 for TCGA and Taylor IMBN, respectively). In contrast, genes with cis-CNAs don't significantly overlap with prostate cancer genes (Fisher's exact test p= 1.0 and 0.3 for TCGA and Taylor IMBN, respectively). ETS-related gene (*ERG*) is an oncogene whose activation is one of the most common oncogenic alterations in PCa, occurring in over 50% of prostate tumors [28]. *ERG* is one of the key regulators in both IMBNs, and *ERG* has the largest number of direct connections in the TCGA IMBN. The *ERG* subnetworks (Figure 2A and 2B) for the TCGA and Taylor IMBNs consist of 136 and 104 genes, respectively. The two subnetworks overlap significantly with 15 genes in common, excluding *ERG* itself (Fisher's exact test *p* = 1 × 10^−10^). When compared with known biological pathways, the “neuronal system” is the most enriched pathway in both subnetworks (Fisher's exact test p=6e-3 and 0.01 for TCGA and Taylor IMBN, respectively) (Supplementary Table S4). It has been shown that *ERG* increases expression of neurotransmitter reporters [29] and *TMPRSS2-ERG* fusion blocks neuroendocrine cell differentiation to allow prostate cancer proliferation [30]. *ERG* activation/overexpression is mainly due to fusion with regulatory sequences of the androgen receptor related prostate cancer genes, predominantly *TMPRSS2*. The *TMPRSS2-ERG* fusion is a well-known PCa driver alteration [13]. The *TMPRSS2-ERG* gene fusion signature identified by comparing PCa patients with and without the fusion [31] was significantly enriched in the *ERG* subnetworks (Figures 2A and 2B, Fisher's exact test p= 1 × 10^−9^ and 7 × 10^−5^ for TCGA and Taylor, respectively). For instance, *HDAC1*, which is associated with the *TMPRSS2-ERG* gene fusion [32], is in the *ERG* subnetwork extracted from TCGA IMBNs (Figure 2A). HDACs play major roles in PCa progression [33]. *ERG* activation results in a large transcriptional response, which is reflected in our IMBNs. More specifically, we varied the specificity of *ERG* subnetworks by including more genes distantly connected to *ERG* in the network (1-specificity=number of genes in a subnetwork/number of genes in an IMBN), and calculated the sensitivity of each *ERG* subnetwork overlapping with the *TMPRSS2-ERG* gene fusion signature (sensitivity=number of genes in the overlap/number of genes in the subnetwork), resulting a significant receiver-operating characteristic (ROC) curve (Figure 2C). The network accuracy is defined as a partial area under the curve (AUC) in the ROC plot with specificity>90%. IMBNs have significantly higher accuracies than random permutated networks and IMBNs with CNA overlap better with the *TMPRSS2-ERG* gene fusion signature (p=0.002 for Taylor IMBN without CNA, and p<0.001 for the other three networks estimated based on 1000 permutations).

*AR* is one of most critical effectors in prostate cancer development and progression. Ligand-activated ARs bind to DNA of target genes and induce their transcription. The transcriptional activity of *AR* is affected by multiple co-regulators that influence a number of functional properties of *AR* [34]. *AR* expression level itself may not correlate well with its transcriptional activity. Instead of testing whether *AR*'s network neighborhoods are enriched for AR signature genes, we searched through IMBNs and identified genes whose neighborhoods are enriched for AR signature genes. The top candidate is *DHCR24*, a FAD-dependent oxidoreductase involved in cholesterol biosynthesis (Fisher's exact test *p* = 3 × 10^−10^, its subnetwork in TCGA IMBN is shown Supplementary Figure S2A). The *DHCR24* subnetwork in the Taylor IMBN is also enriched for *AR* signature genes (Supplementary Figure S2B, Fisher's exact test *p* = 8 × 10^−67^). *DHCR24* is shown to be regulated by AR in prostate cancer [35].

In summary, our analysis demonstrates that IMBNs recapitulate important known biological interactions in PCa.

### Identification of subnetworks associated with BCR of PCa

**Figure 3:**
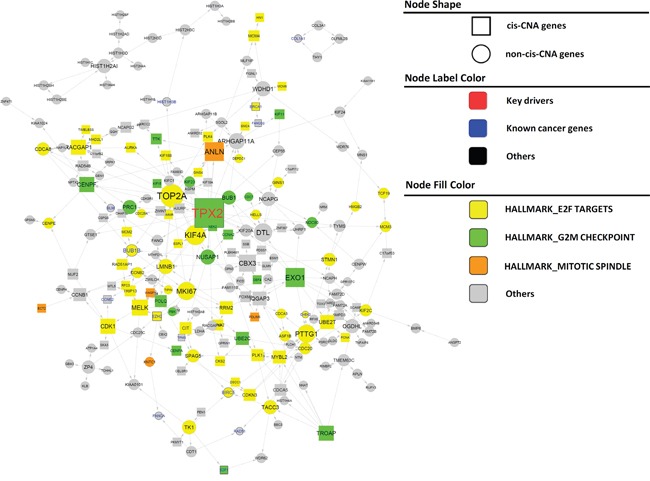
Subnetworks positively associated with BCR in PCa Nodes of varied colors represent different pathways enriched in the subnetwork. Genes known to be cancer related are labeled in larger font size with blue color (nodes with thicker boarder). Key driver genes are labeled in larger front size with red color. Square nodes represent genes regulated by cis CNA. Edges directly associated with key drivers and NLGN4Y are colored blue and red, respectively.

**Figure 4:**
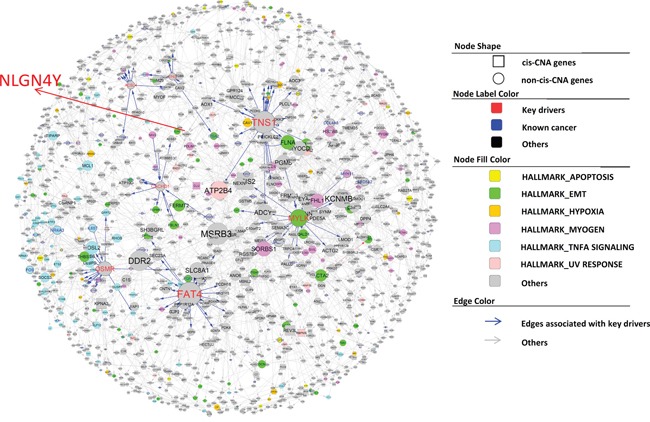
Subnetworks negatively associated with BCR in PCa The node annotation is the same as in Figure 3.

After demonstrating that our IMBNs are able to recapitulate previously recognized markers of PCa, we next tried to use these networks to identify new molecular mechanisms of PCa progression. Based on BCR data in the Taylor dataset, we identified an initial list of 189 and 378 genes positively and negatively associated with BCR (Cox regression p-value <0.01 after multiple testing correction). Using the same threshold, there was no gene associated with BCR in the TCGA PRAD data set, which is likely due to shorter follow-up time in TCGA compared to the Taylor dataset (Table 1). Nevertheless, BCR-associated genes identified in the Taylor dataset rank significantly higher in their associations with BCR in the TCGA PRAD data set compared to the rest of the genes (Wilcoxon rank sum test p= 1 × 10^−47^ and 5 × 10^−69^ for positive and negative associated BCR genes, respectively), indicating similarity between the two datasets. As expected, the BCR genes are significantly enriched for previously identified prostate cancer genes (Supplementary Table S3) (Odds ratio=9.7, Fisher's exact test *p* = 1 × 10^−19^). We then projected BCR genes onto IMBNs and constructed BCR subnetworks (Supplemental Methods). Compared to the initial BCR gene list, the BCR subnetworks contain almost twice as many genes (a total of 989 genes for Taylor IMBN and 1003 for TCGA IMBN). The BCR subnetworks are even more significantly enriched for known prostate cancer genes (Odds ratio=17.0 and 11.0, Fisher's exact test p= 2 × 10^−48^ and 5 × 10^−31^ for Taylor and TCGA IMBN, respectively). The two BCR subnetworks derived from TCGA and Taylor IMBN are similar in terms of enriched pathways (Supplementary Table S5). We combined the two subnetworks together in further analyses. The positive BCR subnetwork (Figure 3) is enriched for the cell cycle related pathways (Supplementary Table S5), such as G2M checkpoint, E2F targets, and mitotic spindle. The negative BCR subnetwork (Figure 4) is enriched for the biological processes TNFα signaling, EMT transition, myogenesis, and apoptosis pathways (Supplementary Table S5).

There are 12 and 63 key regulators for the positive and negative BCR subnetworks, respectively. However, many key regulators are close to each other in the subnetworks. To find distinct key regulators and regulated biological pathways, we filtered out key regulators that are directly regulated by other key regulators with higher degree, resulting only one distinct key driver, *TPX2*, and 7 distinct key drivers, *MYLK, FAT4, TNS1, OSMR, TP63, ACSL4*, and *CACHD1*, for positive and negative BCR subnetworks, respectively (Figure 3 and Figure 4). For a detailed description and discussion on these key drivers, please refer to Discussion.

**Figure 5:**
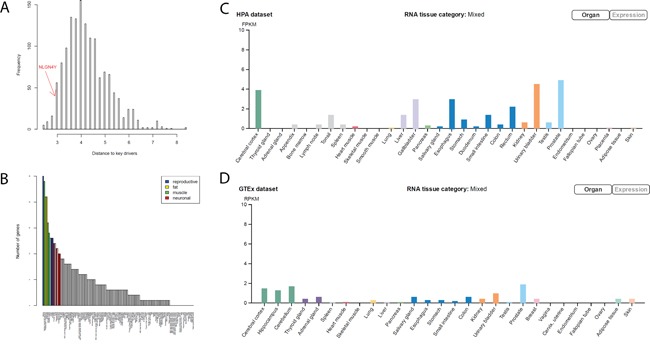
*NLGN4Y* as a common downstream gene (mediator) of the 5 key drivers in the negative BCR network **A.** The distribution of the average shortest distance to the 5 key drivers. *NLGN4Y* is among the top 10% of genes closest to the 5 key drivers, noted as common downstream genes of the 5 key drivers. **B.** The number of common downstream genes preferentially expressed in each tissue, suggesting that common downstream genes are preferentially expressed in reproductive, muscle, fat, and neuron related tissues. **C and D.** *NLGN4Y*, which is expressed highest in prostate tissue, is preferentially expressed in neuron-related tissues according to the HPA dataset (C) and the GTEX dataset (D).

Most of the key regulators (*MYLK, FAT4, TNS1, TP63*, and *CACHD1*) in negative BCR networks are involved in closely related biological processes such as myogenesis, epithelial-mesenchymal transition, and apical junction/surface. To understand how the signals from these key drivers are integrated together, we tried to identify a set of common mediators [36] or signal integrators of these key drivers. There are multiple approaches for identifying common mediators, such as random walk [37], PageRank-liked propagation [38], and shortest path-based methods. Here we used the shortest path method for identifying common mediators as following: for each gene in the negative BCR network, we calculated its mean shortest distance to the above 5 key regulators (the distribution shown in Figure 5A). We defined the top 10% genes with the shortest distance as the common downstream genes of key regulators. When analyzed tissue expression of these common downstream genes using human tissue atlas (see for Methods details), we found that the common downstream genes are preferentially expressed in fat (adipocytes and subcutaneous preadipocytes), muscle-related tissues (cardiac and skeletal muscle), reproductive specific tissues (uterus and ovary), neuron-related tissues (superior cervical ganglion, prefrontal cortex, and caudate nucleus), and conjunctiva (Figure 5B). Adipocytes have been shown to drive prostate cancer progression in many studies [39]. Genes preferentially expressed in reproductive specific tissues among the common downstream genes may be the result of variations of estrogen receptor regulated genes. The connection to neuron-related tissues is unexpected. It has been shown that the severity of prostate cancer is associated with increase of nerve cells in and around the tumor [40]. However, we found genes preferentially expressed in neuronal cells were actually down-regulated in severe prostate cancers, including *APP*. Particularly, among the 12 common downstream genes preferentially expressed in prefrontal cortex (Figure 5B), *NLGN4Y* ranks the first in terms of the mean shortest distance to the 5 key drivers. *NLGN4Y* encodes a protein involved in synaptic adhesion in neurons, and its expression level is the highest in prostate and followed by neuronal system according to two tissue gene expression studies [41, 42] (Figure 5C and 5D).

### Lower expression of *NLGN4Y is associated with higher grade PCa and higher risk for BCR*

**Figure 6:**
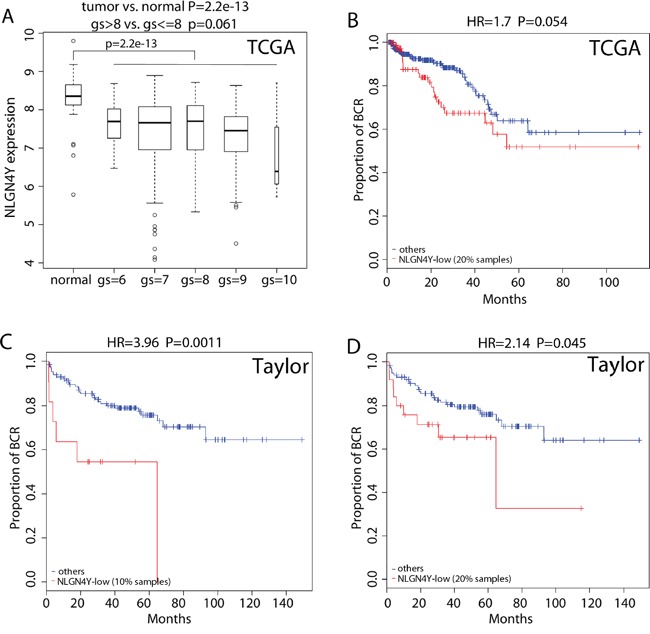
*NLGN4Y* is a regulator of BCR **A.** Boxplot of *NLGN4Y* expression in PCa samples with different gleason scores (gs) as well as normal prostate samples. **B-D.** Kaplan-Meier curves of BCR for patients with lower and higher expression of *NLGN4Y* in the TCGA dataset (B) and the Taylor dataset using different splitting of low and high (C and D). P-values were calculated by log rank test.

*NLGN4Y* expression is significantly lower in tumor tissues compared to the normal tissues in the TCGA dataset (Figure 6A, p-value=2×10^-13^). *NLGN4Y* expression is also lower in high grade tumors (Gleason score >8) compared to lower grade disease (Gleason score <=8) with borderline significance (Figure 6A, p-value=0.061). Compared with *NLGN4Y* expression in the normal tissues, 20.0% tumor samples in the TCGA set expressed significantly lower level of *NLGN4Y* (p<0.01 under the normal distribution with mean and standard deviation estimated from the normal tissues). The *NLGN4Y*-low group was associated with higher risk for BCR in the TCGA set (Figure 6B, HR=1.7 and p=0.054). Since the Taylor dataset doesn't consist of any normal samples, we used the distribution of *NLGN4Y* expression in the TCGA data set as a reference for categorizing its expression in the Taylor data set. Given the Taylor data containing more patients of lower disease grade than those in the TCGA dataset, we assume the percentage of *NLGN4Y*-low patients is less than 20%, the percentage in the TCGA dataset. Figure 6C and 6D show the survival curves when the percentage cutoff is set to be 10% and 20%. In both cases, the *NLGN4Y*-low group was associated with significantly higher risk for BCR (HR=3.96 and 2.14, p=0.0011 and 0.045, respectively).

### NLGN4Y expression is undetectable in more than half of PCa cell lines tested

*NLGN4Y* is specifically expressed in prostate and neuron-related tissues (Figure 5C and 5D). To test whether the above association is due to higher tumor purity in tumors of higher grade and/or due to *NLGN4Y* expression change in prostate cancer cells, we checked *NLGN4Y* transcript expression by qPCR in a panel of benign and malignant prostate cell lines. The probe is specific to *NLGN4Y* and does not detect *NLGN4X* mRNA, which shares 97% sequence homology with *NLGN4Y*. The results show that *NLGN4Y* is expressed in primary normal prostate epithelial cells (hPrEpiC), primary normal prostate fibroblast cells (HPrF) and prostatic intraepithelial neoplasia cells (PIN), but its transcript could not be detected in 8 of 11 cancer cell lines (i.e. VCaP, LNCaP 104S, MDA-PCa-2b, DU145, PC-3, PC-3M, ARCaPM and LNCaP 104R2) (Supplementary Figure S3). Due to lack of a NLGN4Y-specific antibody, protein expression in these cell lines could not be evaluated. There is no association between androgen sensitivity and *NLGN4Y* expression status in these cell lines (p-value=0.85, chi square test).

### NLGN4Y negatively controls cell proliferation

**Figure 7:**
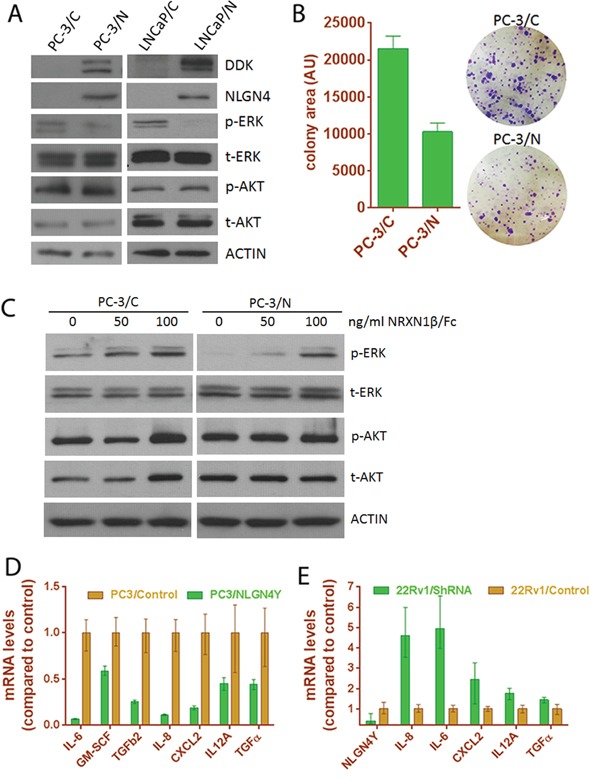
Expression of *NLGN4Y* decreases cell proliferation and pro-inflammatory cytokine expression **A.** *NLGN4Y* expression decreased ERK phosphorylation in PC-3 and LNCaP cells. **B.** *NLGN4Y* expression decreased colony formation of PC-3 cells. **C.** Treatment of PC-3 cells with decoy receptor NRXN1β/Fc for 1 hour abolished the effect of *NLGN4Y* expression on ERK phosphorylation. **D.** *NLGN4Y* expression downregulated pro-inflammatory cytokine expression in PC-3 cells. **E.** *NLGN4Y* shRNA upregulated these cytokine expression in 22Rv1 cells.

To further understand the role of *NLGN4Y* in PCa progression, we ectopically expressed the protein in NLGN4Y-null PC-3 cells and in LNCaP cells, in which low endogenous *NLGN4Y* mRNA can be detected by qPCR at high amplification cycles, but protein expression is hardly detectable by western blot (Supplementary Figure S4A). Cell lines stably expressing NLGN4Y (PC-3/N and LNCaP/N) and vector control cells (PC-3/C and LNCaP/C) were generated through viral transduction followed by puromycin selection. The protein expression of exogenous NLGN4Y was confirmed by Western blot using an antibody against the DDK tag (Figure 7A) and an additional NLGN4 antibody (R&D Systems) which recognizes both NLGN4X and NLGN4Y (Figure 7A). We derived monoclonal PC-3 cells (namely C9 and C15) which express relatively high level of NLGN4Y (Supplementary Figure S5A).

PC-3/N cells grow slower than vector control, as reflected by significantly decreased colony sizes in a clonogenic assay using polyclonal PC-3/N cells (Figure 7B). Cell proliferation data obtained from monoclonal PC-3/N cells (Supplementary Figure S5B) also support this observation. Clone C9 and C15 displayed a longer doubling time compared to vector control. Because these monoclonal cells expressed higher NLGN4Y levels than polyclonal cells (Supplementary Figure S5A), the inhibitory effect on cell proliferation appears to be more pronounced. We next examined the activation of the ERK pathway in these cells by Western blot (Figure 7A). *NLGN4Y* expression significantly diminished phosphorylation of ERK, which is a major cellular pathway involved in regulation of cell proliferation. AKT phosphorylation was not changed by NLGN4Y; however due to the fact that both cell lines harbor PTEN loss, the effect of NLGN4Y on AKT pathway remains to be further characterized. To further support the observation, we knocked down *NLGN4Y* transcript expression by 80% using shRNA in 22Rv1 cells and observed enhanced ERK phosphorylation and colony formation (Supplementary Figure S6). Thus, NLGN4Y overexpression negatively regulates ERK phosphorylation.

NLGNs are characterized as ligands to β-neurexins (NRXNs), another group of membrane adhesion proteins located in the pre-synaptic neuron [43]. To examine if the inhibitory effect of NLGN4Y on ERK is mediated through membrane NRXN receptors in prostate cancer cells, we treated PC-3 cells with soluble NRXN1β/Fc (R&D Systems) which served as a decoy receptor to inhibit NLGN4Y binding to membrane NRXNs [44]. We found that NLGN4Y overexpression inhibited p-ERK in the absence of NRXN1β/Fc; however, this inhibition can be relieved by treating PC-3/N cells with increasing concentrations of soluble NRXN1β/Fc (Figure 7C). 100 ng/ml NRXN1β/Fc could almost completely abolish the effect of NLGN4Y overexpression on p-ERK, suggesting that soluble NRXN1β/Fc could prevent the binding of NLGN4Y to membrane bound NRXNs and inhibit the effect of NLGN4Y overexpression on ERK phosphorylation. In PC-3/C control cells, NRXN1beta/Fc showed a slight effect on p-ERK, which could be due to the effect of soluble NRXN1β/Fc on other NLGNs (such as NLGN2). Similar observation was made in LNCaP cells (Supplementary Figure S4). But the details of this interaction remain to be further elucidated.

Previously, inflammatory cytokines such as IL-6 and IL-8 have been reported to stimulate the growth of prostate cancer cells, especially androgen-independent prostate cancer cells [45–49]. To understand if *NLGN4Y* regulates cell proliferation by altering the expression of these cytokines, we examined the expression of several cytokines by qPCR and found that mRNA of IL-6, IL-8, GM-CSF and several other inflammatory cytokines were significantly downregulated in PC-3/N cells (Figure 7D). Since LNCaP cells have very low levels of endogenous IL-6 and IL8 expression, the negative effect of NLGN4Y on the cytokine expression could not be assessed accurately. However, when we knocked down *NLGN4Y* in 22Rv1 cells, the mRNA levels of these cytokines were upregulated to differing extents, with IL-6 and IL-8 displaying the most significant changes (Figure 7E). The results altogether show that NLGN4Y is involved in the regulation of inflammatory cytokine expression. In addition, we also found that the expression of several neurotropic factors was regulated by NLGN4Y (Supplementary Figure S7), the impact of which on these prostate cancer cells is unknown.

### NLGN4Y expression decreased cell migration through modifying small Rho GTPase activities

**Figure 8:**
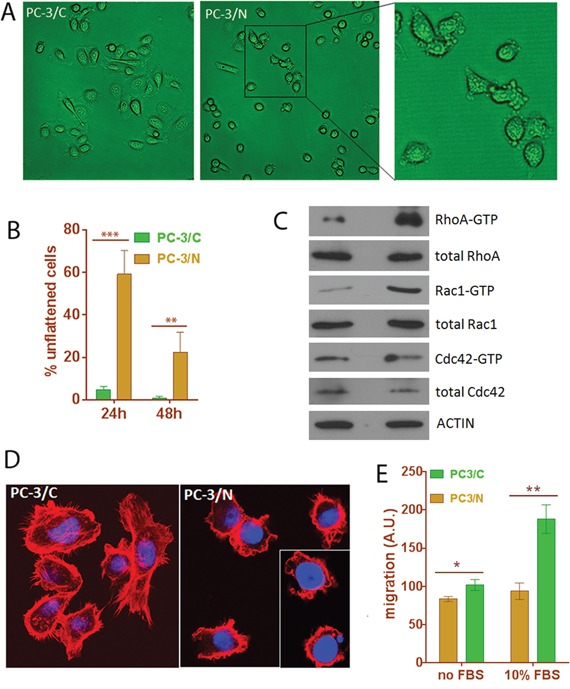
Expression of *NLGN4Y* in PC-3 leads to changes in cell morphology, cell motility and small Rho GTPase activities **A** and **B.** *NLGN4Y* expression induced prolonged membrane blebbing stage during attachment to culture vessel. **C.** *NLGN4Y* expression caused changes in small Rho GTPase activities. **D.** *NLGN4Y* expression induced abnormal F-actin organization as shown by phalloidin-rhodamine staining. **E.** *NLGN4Y* expression significantly decreased cell migration in a transwell migration assay using serum as a chemo-attractant. *: p<0.05; **: p<0.01 and ***: p<0.001.

Since NLGN4Y is a well-known membrane protein involved in synaptic membrane adhesion [43, 44, 50], we speculated that it may also play roles in cancer cell adhesion and mobility. Indeed, we observed that PC-3/N cells displayed abnormalities in cell attachment. While control cells appeared flat and well-attached 24 hours after seeding, >50% of PC-3/N cells were still round, loosely attached to culture vessel and displayed extensive membrane blebs (Figure 8A and 8B). Forty-eight hours after seeding, ∼80% PC-3/N cells eventually appeared fully attached and the remaining cells were still showing membrane blebbing. The monoclonal cells C9 and C15, which expressed higher levels of NLGN4Y (Supplementary Figure S5A), spent longer in cell attachment (Supplementary Figure S8A). Even 48 hours after seeding, >50% of cells were still in the stage of membrane blebbing. Membrane blebbing is an early step in cell attachment but is also observed in multiple cellular processes including early apoptosis [51, 52], we ruled out the possibility that cells were undergoing apoptosis by negative results in an annexin-V/PI apoptosis assay (data not shown). It is more likely that PC3/N cells display a prolonged membrane blebbing stage during cell attachment.

Although membrane blebbing has been associated with various conditions of cells, i.e. apoptosis, cytokinesis, cell spreading viral infection and cell motility [51, 52], activation of Rho-GTPase underlies virtually all types of membrane blebbing [52–55]. To further examine if NLGN4Y-induced extensive membrane blebbing is mediated through abnormal small Rho GTPase activity, we performed small Rho GTPase assays. The result showed that PC-3/N cells had elevated RhoA and Rac1 activity but unchanged Cdc42 activity (Figure 8C), suggesting that a disturbance in small GTPase activities may contribute to the prolonged membrane blebbing stage during cell attachment.

The small Rho-GTPase proteins, Cdc42, Rac and Rho, coordinate cell migration by regulating cytoskeletal reorganization [52]. We next examined the distribution of filamentous (F) actin using phalloidin-rhodamine staining in these cells. In PC-3/C cells, actin was highly polymerized into stress fibers and well-defined filopodia and lamellipodia (Figure 8D). However, in PC-3/N cells, few stress fibers or lamellipodia were observed and the number of filopodia was dramatically decreased (Figure 8D and Supplementary Figure S8B). Instead, actin staining was prominent around the plasma membrane to form extensive membrane blebs, altogether suggesting NLGN4Y expression induced abnormal F-actin organization.

Abnormal cytoskeleton organization often have a negative impact on cell motility [51]. We next performed a transwell migration assay to examine whether abnormal cytoskeleton organization was accompanied by any defect in cell migration. The migration was assessed in the absence and presence of FBS. In FBS-free condition, random cell movement was measured. In FBS-containing condition, chemoattractant stimulated directional cell migration was measured. PC-3/N cells showed slightly decreased random cell movement in the absence of FBS; however directional cell migration induced by FBS was significantly down by ∼50% in PC-3/N cells compared to PC-3/C (p-value<0.001), indicating that NLGN4Y overexpression negatively impacted prostate cancer cell migration (Figure 8E). The same result was obtained for the monoclonal C9 and C15 cells in a wound healing assay (Supplementary Figure S8C).

In summary, overexpression of NLGN4Y upregulated RhoA and Rac1 activities, which lead to abnormal F-actin organization and decreased cell migration.

## DISCUSSION

In this study, we reconstructed IMBNs by integrating gene expression and CNA profiling data from two published studies and showed that CNA data integration improved IMBN accuracy. The reconstructed IMBNs recapitulated the known biology of PCa. We constructed subnetworks associated with BCR and identified multiple key regulators in the BCR subnetworks, such as *TPX2, MYLK, FAT4, TNS1, OSMR, TP63, ACSL4*, and *CACHD1*. We analyzed the correlations of the 8 key regulators with other clinical variables known to have prognostic power in Taylor's dataset, i.e., Gleason score, T stage and N stage. As shown in the Supplementary Figures S9-S11, all 8 key drivers are significantly correlated with N stage, 5 of them are significantly correlated with Gleason score and 2 are significantly correlated with T stage (F test p<0.05). We then tested the residual association between each key driver gene and BCR when the above three clinical variables are considered in the multivariable cox regression model. 5 of the key drivers become insignificant (Wald test p>0.1), likely due to their correlation with the three clinical variables or insufficient power for multi-variable analysis given the sample size. However, 3 of them remain significant or marginally significant, i.e., OSMR (p=0.026), TP63 (p=0.028) and CACHD1 (p=0.081), suggesting they carry additional prognostic power. We note that the main purpose of this study is not to find genes carrying prognostic power, but more to understand the biological mechanisms and pathways underlying prostate cancer progression.

Literature and previous studies highlight the potential pathways through which the 8 key regulators affect cancer progression. *TPX2* is a microtubule associated protein, which is required in forming mitotic spindles during cell cycle. The *TPX2* subnetwork (Figure 3) significantly enriched for the biological process G2M checkpoint (Fisher's exact test *p* =3.1 × 10^−42^). *TPX2* overexpression is a biomarker of poor prognosis in brain, breast, colorectal, and lung cancers [56]. *TPX2* knockdown reduced prostate-specific antigen (PSA) expression in PCa cell lines, indicating that *TPX2* regulates AR signaling in PCa [57], and induced cell cycle arrest, apoptosis, and the inhibition of cell proliferation and invasion in multiple other cancers [58, 59]. *MYLK* (also known as *MLCK*), myosin light chain kinase, is the top key regulator for the negative BCR subnetwork. It is shown as the best discriminator for prostate cancer [60]. *MYLK* subnetwork (Figure 4) is significantly enriched for genes involved in the pathways myogenesis and epithelial-mesenchymal transition (Fisher's exact test p= 3.2 × 10^−8^ and 7.5 × 10^−4^ respectively). *MYLK* is down-regulated by androgens in human prostate cancer cells [61], and acts as a central mediator of migration, proliferation and invasion of prostatic adenocarcinoma cell line [62]. *FAT4*, a member of the protocadherin family, plays a role in regulating planar cell polarity and inhibit neuroprogenitor cell proliferation and differentiation. *FAT4* subnetwork (Figure 4) is enriched for the biological process epithelial-mesenchymal transition (Fisher's exact test *p* =1.6 × 10^−8^). *FAT4* is identified as a tumor suppressor gene in breast cancer [63], and lung cancer only in males [64]. The *TNS1* subnetwork (Figure 4) is enriched for the biological pathway, myogenesis (Fisher's exact test 4.9 × 10^−13^). *TNS1* encodes tensin 1, which localizes to focal adhesion and acts as a tumor suppressor in PCa [65]. Expression of *TNS1* decreased the migration and invasion of triple-negative breast cancer cells [66]. *OSMR*, oncostatin M receptor, is a member of the type I cytokine receptor family. The OSMR subnetwork (Figure 4) is enriched for the biological process TNF signaling via NFKB (Fisher's exact Test *p* =4.1 × 10^−20^). OSMR forms a heterodimer with IL6ST and plays a role in PCa progression [67]. *TP63* is a member of the p53 family of transcription factors. The TP63 subnetwork (Figure 4) is enriched for genes involved in apical junction (Fisher's exact test 1.3 × 10^−4^). Low expression of *TP63* is associated with PCa progression [68, 69]. ACSL4 is an isozyme of the long-chain fatty-acid-coenzyme A ligase family. The *ACSL4* subnetwork (Figure 4) is not enriched for any hallmark biological process. *CACHD1*'s function is not clear. Mouse homolog *Cachd1* is predicted to involve in the biological process establishment of localization. The *CADHD1* subnetwork (Figure 4) is enriched for the biological process forming apical surface (Fisher's exact test *p* = 6.8 × 10^−4^), consistent with its predicted function in mouse.

To further validate the constructed BCR sub-networks in their involvement in BCR, we analyzed a third independent dataset [70]. The dataset contains 281 cases from the population-based Swedish-Watchful Waiting cohort. The cohort consists of men with localized prostate cancer (clinical stage T1-T2, Mx, N0), which are quite different from patient characteristics in the TCGA or Taylor datasets. Their gene panel only contains 6100 genes. Despite the obvious differences, we observed significant overlap of BCR genes in this dataset and our BCR network. For instance, among the total 6100 genes profiled, 6.7% (412) are negatively correlated with BCR in this dataset (p<0.05). If focused on genes within our negative BCR sub-networks, 16.1% are negatively correlated with BCR (Odds Ratio=3.3, p= 1 × 10^−21^). The percentage further increases to 25.7% (Odds Ratio =5.0, p= 7 × 10^−10^) when only common downstream genes of the key regulators are considered.

**Figure 9:**
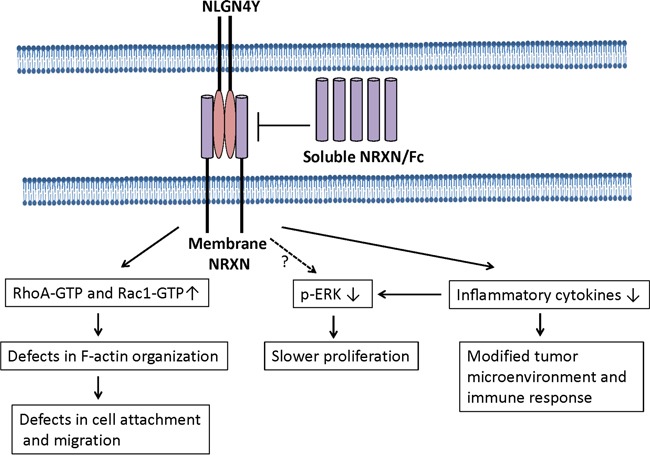
A model to illustrate NLGN4Y signaling in prostate cancer cells NLGN4Y binds to membrane-bound NRXNs to regulated small Rho-GTPase activities, inflammatory cytokine expression and ERK phosphorylation. Soluble NRXN/Fc blocks to the binding of NLGN4Y to membrane NRXN, thus inhibiting NLGN4Y signaling. Currently, it is not known if NLGN4Y downregulates ERK phosphorylation directly and/or indirectly through controlling inflammatory cytokine expression (eg. IL-6 and IL-8). NLGN4Y expression elevates RhoA-GTPase and Rac1-GTPase activities, which cause abnormal in F-actin organization and subsequently decrease cell migration.

We further showed that key regulators for biological processes myogenesis and epithelial-mesenchymal transition regulated a common set of downstream genes and identified *NLGN4Y*, a gene highly expressed in neurons, as a novel regulator of biochemical recurrence of prostate cancer. *NLGN4Y* expression is lost in more than half of the PCa cell lines examined, while restoration of *NLGN4Y* led to decrease in cell proliferation, inflammatory cytokine expression and cell migration (Figure 9). Our analysis showed that lower expression of *NLGN4Y* is associated with higher risk for BCR in both TCGA and Taylor datasets. Our studies also suggest that *NLGN4Y* may negatively impact PCa metastasis potential through regulating cytoskeleton organization and cell migration; thus it is a novel mediator of PCa prognosis.

NLGNs are a family of transmembrane adhesion proteins expressed in the postsynaptic neurons [71]. They are characterized as ligands to β-neurexins (NRXNs), another group of membrane adhesion proteins located in the pre-synaptic neuron. The interaction between NLGNs and NRXNs is essential for stabilization of synaptic contacts and vesicle clustering and maturation [43]. In our study, addition of soluble NRXN1β as a decoy receptor abolished the effect of NLGN4Y expression on ERK, indicating that the effect of NLGN4Y is mediated at least through NRXN1β. So far, there are 5 NLGN genes and 3 NRXN genes identified in human. Each gene product has overlapping as well as unique influences on synaptic transmission [72]. Our study demonstrated an initial link between a synaptic adhesion protein expression and prostate cancer recurrence. In future study, the role of and complicated interaction of these NLGNs and NRXNs in prostate and other cancer remain to be illustrated.

Inflammatory cytokines such as IL-6 and IL-8 have been reported to stimulate the growth of prostate cancer cells especially androgen-independent prostate cancer cells [45–49]. Our study showed that *NLGN4Y* knockdown by shRNA significantly upregulated several important pro-inflammatory cytokine expression in 22Rv1 cells, whereas restoration of *NLGN4Y* expression in PC3 cells dramatically downregulated them. Thus NLGN4Y will have a negative effect on ERK signaling indirectly through downregulation of IL-6 and IL-8 production (Figure 9). It remains unknown if NLGN4Y will direct regulated ERK signaling independent of IL-6 and IL-8 production. In addition to its role in controlling cell growth and motility, loss of *NLGN4Y* expression in cancer cells may lead to a more inflammatory tumor microenvironment. The upregulated cytokines may modify host immune response and activate angiogenic programs to promote cancer progression [73, 74]. To date, the pathways leading to decreased cytokine upregulation by NLGN4Y remain unknown.

Restoration of *NLGN4Y* expression in PC-3 cells rendered prolonged membrane blebbing during cell attachment. Although membrane blebbing has been associated with various conditions of cells, i.e. apoptosis, cytokinesis, cell spreading viral infection and cell motility [51, 52], activation of Rho-GTP and its effector kinase ROCK underlies virtually all types of membrane blebbing [52–55]. Our observation that *NLGN4Y* expression altered Rho GTPase activities and subsequently caused extensive membrane blebbing is consistent with reports in the literature. Our study is the first to link *NLGN4Y* to the regulation of Rho GTPase activity. Whether the same interaction exists in neurons remains an interesting question. Given the pivotal role of Rho in regulating axonal outgrowth and cell migration during neural development [75–78], it is likely that *NLGN4Y* also regulates neuron migration and growth in a Rho-dependent way.

NLGNs and NRXNs are well-characterized synaptic cell adhesion molecules [43]. Mutations in these genes are associated with cognitive diseases such as schizophrenia and autism spectrum disorders [43, 79]. However, the function of NLGNs outside of the nervous system has been rarely described. Bottos et al reported that NRXNs and NLGNs are produced and processed by endothelial and vascular smooth muscle cells throughout the vasculature [80]. *NLGN4* mRNA is found with the highest relative expression in heart tissue with lower expression levels detected in liver, skeletal muscle and pancreas [81]. This widespread tissue expression indicates potential novel functions of this synaptic protein outside of the CNS. *NLGN4Y* is not the only neuron-derived protein that plays a role in cancer. Plexin B1, the cellular receptor for semaphorins [a family of soluble and membrane associated proteins that play a critical role in axonal guidance [82]], is often mutated or overexpressed in primary and metastatic PCa [83]. The mutations hinder Rac and R-Ras binding and R-RasGAP activity, resulting in an increase in cell motility, invasion, adhesion, and lamellipodia extension. The roles these neuron-derived proteins play in PCa remain an interesting question. PCa is well-known for the importance of perineural invasion, a phenomenon of cancer spreading to the space surrounding a nerve [84, 85]. The prostate is densely innervated with hypogastric and pelvic nerves that plays an important role in regulating the growth and function of the prostate gland [86]. Being a common component of the tumor microenvironment, nerves and nerve-derived substances may actively participate in normal, benign and malignant PCa growth. Studies have shown that autonomic nerve development may contribute to PCa progression [87]. Chemical or surgical sympathectomy inhibited prostate tumorigenesis in animal models. In our study, several neurotropic factors were negatively regulated by *NLGN4Y* (Supplementary Figure S7). Thus, it remains an interesting question whether *NLGN4Y* loss has any effect on the surrounding nerves in PCa and how this interaction will impact tumor progression *in vivo*.

In summary, our preliminary studies *in vitro* support the role of *NLGN4Y* as a negative regulator of PCa progression. However, we recognize that there are limitations of cell line models which are incapable of recapitulating complicated tumor-host interactions. *NLGN4Y* may display a more profound role of regulating immune response and nerve cell activity by controlling inflammatory cytokine and neurotropic factor expression. Ultimately, these possibilities remain to be revealed *in vivo*.

## MATERIALS AND METHODS

### Constructing IMBNs for prostate cancer by integrating gene expression and CNA data

Two prostate cancer data sets were used in the study, the TCGA prostate adenocarcinoma (PRAD) study [15] and Taylor prostate cancer study [21]. For the TCGA PRAD dataset, the gene expression and gene-based CNA data were downloaded from the TCGA data portal (https://tcga-data.nci.nih.gov/tcga/). For the Taylor dataset, gene expression and CNA data were downloaded from the GEO repository with accession numbers GSE21034 and GSE21035. Please see Supplemental Data for detailed network construction and analysis.

### Cell culture and reagents

The human PCa cell lines LNCaP FGC, PC-3, DU145 and MDA-PCa-2b were acquired from ATCC. PC3M and PIN cells were obtained from Dr. Goutham Narla (Mount Sinai School of Medicine, NY). 22Rv1 and LAPC-4 cell lines were obtained from Dr. Liying Zhang (Memorial Sloan-Kettering Cancer Center, NY). LNCaP 104S and 104R2 cell lines were provided by Dr. Shutsung Liao (Univ. of Chicago, IL). ARCaPM cell line was a gift from Dr. Josep Domingo-Domenech (Mount Sinai School of Medicine, NY). Human normal prostate epithelial cells (HPrEpiC) and human prostate stromal fibroblasts (HPrF) were purchased from the ScienCell and cultured in the Fibroblast Medium (ScienCell). All cell lines were maintained in complete growth medium with Pen-Strep Solution (Gemini Bio-Products) in a humidified incubator with 5% CO_2_ at 37°C. P-ERK, t-ERK, p-AKT and t-AKT antibodies were obtained from Cell Signaling Technology and actin antibody was purchased from Sigma. DDK antibody is from Origene and NLGN4 antibody is from R&D Systems.

We obtained the *NLGN4Y* cDNA with a DDK tag from OriGene Technology (Rockville, MD) and then fully sequenced the gene insert. DDK-tagged *NLGN4Y* cDNA was subcloned into pBabe-puro retroviral vector through BamHI and ECoR1 sites using PCR primers (NLGN4Y-F: TCGTCGACTGGATCCGGTA; NLGN4Y-R-ECORI: gaGAATTC GTTTAAACCTTATCGTCGTCATCC). Vector control cell lines (PC-3/C and LNCaP/C) and NLGN4Y expressing cells (PC-3/N and LNCaP/N) cells were generated by retroviral transduction followed by puromycin selection (1 μg/ml for PC-3 cells, and 4 μg/ml for LNCaP cells).

### Realtime qRT-PCR analysis

Total cellular RNA was isolated by Trizol reagent (Life Technologies) and then used for cDNA synthesis by Superscript III platinum one-step qPCR kit (Life Technologies). Quantitative PCR analysis of mRNA expression was performed with inventory Taqman gene expression assays on ViiA7 realtime PCR instrument (Life Technologies). The gene expression levels were normalized to endogenous control RPLP0.

### Immunoblotting

Cellular protein was harvested by lysing cells in RIPA lysis buffer containing protease inhibitor cocktail (Thermo Scientific), followed by centrifugation at full speed to collect the supernatant. The harvested protein was separated by SDS-polyacrylamide gel electrophoresis and transferred to Immobilon-P membranes (Millipore). Membranes were blocked with 5% nonfat milk or BSA in TBST buffer and incubated with the corresponding primary and secondary antibodies. Protein signals were visualized by Supersignal® West Pico Luminol/Enhancer Solution (Thermo Scientific).

### F-actin staining and confocal imaging

Cells grown in Millicell® EZ SLIDES were fixed in 4% paraformaldehyde solution for 10 min at room temperature (RT) and then incubated in blocking/permeabilizing solution (5% BSA and 0.5% Triton X-100 in PBS) for 30 min at RT. Cells then were stained with phalloidin-rhodamine (Cytoskeleton) for 30 min according to manufacturer instruction at RT. After washing three times with PBS, slides were mounted in DAPI-containing Vectashield® mounting medium (Vector Laboratoryies, Inc., Burlingames, CA). Digital images were obtained with a Leica SP5 DMI inverted confocal microscope.

### Clonogenic assay

2000 cells were seeded evenly into 6-well cell culture plate in triplicates. After 10-14 days of incubation, cells were fixed with 10% formalin and stained with 0.005% crystal violet for 1 hour. Digital pictures were taken and the colony number, size and area of the colonies were analyzed with ImageJ software.

### Cell counting

Five random images of cells were taken under microscope from control and NLGN4Y overexpressed PC-3 cells. The numbers of flattened and unflattened cells were counted manually and the percentages were calculated.

### Small Rho GTPase assays

PC-3/N and PC-3/C cells were grown in 10-cm cell culture dishes in serum free medium. Total protein was harvested using RIPA lysis buffer containing protease inhibitor cocktail. The small Rho GTPase pull down assays were performed using active Rho detection kit (Cell Signaling Technologies), active Rac1 pull-down and detection kit and active Cdc42 pull-down and detection kit (Thermo Fisher Scientific) according to manufacturer instructions.

### Migration assay

Cell migration assay was performed using Corning 96-well transwell insert with 8 μm pores according to manufacturer provided protocol. Transwell insert was not coated with any base membrane extract in the migration assay. Briefly, PC-3 cells were serum starved overnight and 5x10^4^ cells were seeded into the transwell insert containing serum free medium in 5 repeats. The transwell culture insert containing cells was then placed on top of a receiver plate containing complete cell culture medium with 10% FBS for 16-17 hours to allow cells to migrate to the receiver plate side. The cells migrated to the bottom of the transwell insert were dissociated from the insert into receiver plate by cell dissociation solution and stained with Calcium AM fluorescent dye. The fluorescent reading of the receiver plate was obtained on a SpectraMax Me microplate reader. The fluorescent reading of PC-3/C cells were set as 100%.

## SUPPLEMENTAL METHODS










