# Supplementary material for: Constructing Bayesian networks by integrating gene expression and copy number data identifies NLGN4Y as a novel regulator of prostate cancer progression
Source: Oncotarget. 2016 Sep 10;7(42):68688–707. doi: 10.18632/oncotarget.11925 (PMC5356583; doi:10.18632/oncotarget.11925)
Supplement: Supplementary file 1 [file oncotarget-07-68688-s001.pdf]

Constructing bayesian networks by integrating gene expression and copy number data identifies *NLGN4Y* as a novel regulator of prostate cancer progression

SUPPLEMENTAL METHODS

Constructing IMBNs for prostate cancer by integrating gene expression and CNA data

Two prostate cancer data sets were used in the study, the TCGA prostate adenocarcinoma (PRAD) study [1] and Taylor prostate cancer study [2]. For the TCGA PRAD dataset, the gene expression and gene-based CNA data were downloaded from the TCGA data portal (https://tcga-data.nci.nih.gov/tcga/). For the Taylor dataset, gene expression and CNA data were downloaded from the GEO repository with accession numbers GSE21034 and GSE21035. All data are log2 transformed.

It has been shown that CNA contributes most significantly to PCa tumorigenesis and progression [3]. CNA alters expression level of underlying genes directly. We defined cis-CNA genes as genes whose expression levels and their CNAs are significantly correlated. Then, we can decompose expression variance of a gene G into multiple parts as $G \sim cisCNA * R + \varepsilon$ (Supplementary Figure S12), due to its cis-CNA, due to its regulators R, and their interactions.

As searching for an optimal Bayesian network structure is a NP-hard problem, we can only include a limited number of genes with informative gene expression or CNAs as nodes in our network reconstruction procedure. For the TCGA PRAD data set, we selected 8,907 informative genes that were expressed in the tumor tissues (the mean expression levels >5) and whose expression levels varied (the standard deviation >0.5). We also included 3,012 cis-CNAs (p-value <0.01 for the Spearman's correlation between gene expression and CNA after multiple testing correction) as nodes. Similarly for the Taylor data set, we selected 6,955 informative genes with the mean expression levels >4 and the standard deviation >0.35 as well as 157 cis-CNAs as nodes.

The selected gene expression and gene-based CNA profiles were input into a Bayesian network reconstruction software package, RIMBANet (Reconstructing Integrative Molecular Bayesian Network) (4–7). A Bayesian network is a directed acyclic graph in which the edges of the graph are defined by conditional probabilities that characterize the distribution of states of each node given the state of its parents [8]. The network topology defines a partitioned joint probability distribution over all nodes in a network, such that the probability distribution of states of a node depends only on the states of its parent nodes: formally, a joint probability distribution $p(X)$ on a set of nodes X can be decomposed as $p(X) = \prod_i p(X^i \mid \mathrm{Pa}(X^i))$, where $\mathrm{Pa}(X^i)$ represents the parent set of X^i. In our networks, each node represents expression level or CNA of a gene. These conditional probabilities reflect not only relationships between genes, but also the stochastic nature of these relationships, as well as noise in the data used to reconstruct the network.

Bayes formula allows us to determine the likelihood of a network model M given observed data D as a function of our prior belief that the model is correct and the probability of the observed data given the model: $P(M \mid D) \sim P(D \mid M) * P(M)$. We can set a model M's prior probability based on biological knowledge. For genes with cis-CNAs, we assume that the expression variations of these genes were directly affected by their CNAs. To represent the assumption, we set a structure prior $p(cis\text{-}CNA_i \rightarrow G_i) = 1$, which is equivalent to start a searching process with an initial structure with a set of $cis\text{-}CNA_i \rightarrow G_i$ edges instead an empty initial structure. We also assume the cis-CNAs only affected expression levels of their cis genes directly, and any trans effects on other genes were through expression variations of their cis genes. Thus, we set the prior $p(cis\text{-}CNA_i \rightarrow G_j) = 0$ for $i \neq j$.

The number of possible network structures grows super-exponentially with the number of nodes, so an exhaustive search of all possible structures to find the one best supported by the data is not feasible, even for a relatively small number of nodes. We employed Monte Carlo Markov Chain (MCMC) [9] simulation to identify potentially thousands of different plausible network structures, which are then combined to obtain a consensus network (see below). Each reconstruction begins with a null network. Small random changes are then made to the network by flipping, adding, or deleting individual edges, ultimately accepting those changes that lead to an overall improvement in the fit of the network to the data. We assess whether a change improves the network model using the Bayesian Information Criterion BIC [10], which avoids overfitting by imposing a cost on the addition of new parameters. This is equivalent to imposing a lower prior probability $P(M)$ on models with larger numbers of parameters.

Searching optimal BN structures given a dataset is an NP-hard problem. We employed an MCMC method to do local search of optimal structures. As the method is stochastic, the resulting structure will be different

for each run. In our process, 1,000 BN structures were reconstructed using different random seeds to start the stochastic reconstruction process. From the resulting set of 1,000 networks generated by this process, edges that appeared in greater than 30% of the networks were used to define a consensus network. A 30% cutoff threshold for edge inclusion was based on our simulation study [11], where a 30% cutoff yields the best tradeoff between recall rate and precision. The consensus network resulting from the averaging process may not be a BN (a directed acyclic graph). To ensure the consensus network structure is a directed acyclic graph, edges in this consensus network were removed if and only if [1] the edge was involved in a loop, and [2] the edge was the most weakly supported of all edges making up the loop.

Network analysis

1) *The degree of a node* in a network is generally defined as the number of edges connecting the node to other nodes. As connections in constructed IMBNs are sparse, we define the degree of a node in an IMBN as the number of nodes that can be reached from the seed node within two hops. 2) *Key regulator analysis* is aimed to identify genes with high potentials to regulate a large number of genes when perturbed. We use the degree of a node in an IMBN defined above as a measurement of transcriptional regulation potential. Given d as the degree of a node, key regulators are defined as nodes with $d > \bar{d} + 2\sigma(d)$. 3) *A gene's network neighborhood* was defined as genes whose distance to the seed gene is <= k. As the size of a gene's network neighborhood greatly affect the significance of functional annotation of the gene's network neighborhood by enrichment analysis, we aimed to define network neighborhoods resulting to similar sizes. We adjusted k according to the connectivity of each gene so that the defined neighborhood was of similar size for all genes. Particularly, for each gene, we chose the smallest k at which the number of genes in its neighborhood is >=100. 4) *Sub-networks associated with Biochemical Recurrence (BCR):* We first selected genes significantly associated (adjusted P-value < 0.01) with BCR using the Cox regression model, termed as "initial BCR genes". To remove sporadic associations and expand high confident associations, we then projected the initial BCR genes onto the IMBNs for prostate cancer constructed above and identified genes whose neighborhood is significantly enriched (p-value of Fisher's exact test $<10^{-6}$) for the initial BCR genes, which together formed BCR subnetworks. The genes in the BCR subnetworks are termed as "network selected BCR genes" collectively. We constructed sub-networks for genes positively and negatively associated with BCR separately.

Collection of reference network databases

To assess accuracies of reconstructed IMBNs for prostate cancers, we collected interactions from multiple databases, including 36,727 interactions covering 9,205 genes from HPRD database [12], 195,859 high confident interactions covering 12,427 genes from STRING database [13], and 476,891 interactions covering 16,010 genes from HumanNet database [14]. We also collected multiple functional gene sets including 186 KEGG [15] pathways covering 5,267 genes, 50 hallmark gene sets [16] covering 4,386 genes, and 1300 GO [17] annotation sets (sets with size >=200 are excluded) covering 6,942 genes from MsigDB databases [16].

Compilation of cancer genes and high confident prostate cancer related genes

We assembled a list of 152 high confident prostate cancer related genes (Supplementary Table S2). These genes have been identified in at least two previous studies related to prostate cancer (Supplementary Table S3). We also compiled a list of 813 known cancer genes (not restricted to prostate cancer) from cancer gene census [18] and KEGG cancer pathways [15] (Supplementary Table S6).

ERG and AR signatures

A list of 87 *TMPRSS2-ERG* fusion signature genes (comparing PCa patients with and without the fusion) was collected from a previous study [19] (Supplementary Table S7). We also compiled a list of 157 AR signature genes (Supplementary Table S8) from multiple sources including 27 AR transcriptional targets based on experimental perturbation [20] and two curated AR pathway gene sets: PID_AR_PATHWAY (61 genes) and HALLMARK_ANDROGEN_RESPONSE (101 genes) from MsigDB database [16].

Human tissue atlas

Gene expression profiles of 126 primary human tissues were downloaded from http://xavierlab2.mgh.harvard.edu/EnrichmentProfiler/download.html. Given a gene, the preferentially expressed tissues are defined as the top 5 tissues with the highest gene expression level.

Patient's clinical information

For the Taylor's dataset, following radical prostatectomy, patients were followed with history, physical exam, and serum PSA testing every 3 months for the first year, 6 months for the second year, and annually

thereafter. Biochemical recurrence (BCR) was defined as PSA ≥0.2 ng/ml on two occasions. For the TCGA PRAD dataset, all tumor samples in TCGA were from primary tumors prior any treatment. BCR events were reported in the dataset without specific definition of BCR.

In the Taylor's dataset, 21.6% of the patients were documented with clinical metastatic events, 72.7% were documented as "NO" in the metastatic event, and 5.6% were NA. For the TCGA dataset, there was no information about metastatic event.

Patient treatment information

The treatment information is sparse for the Tylor and TCGA datasets. For Tylor' dataset, 9.3% of patients are documented with chemotherapy, and the rest are documented as "NA"; 23.3% are documented with hormone therapy, and the rest "NA"; 16% are documented with radiotherapy, and the rest "NA". In summary., 6% of patients are documented with all three types of therapies, i.e., chemotherapy, hormone therapy and radiotherapy, 10.7% documented with two types of therapies, 9.3% documented with one type of therapy and 74% documented with NA for all three therapies. For TCGA dataset, 7.4% patients are documented as "YES" in adjuvant radiation, 40% as "NO", the rest 52.6% as "Not Available" or "Unknown". As for drug treatment, 11.4% are documented with hormone therapy, 1% with chemotherapy, and the rest with no information.

SUPPLEMENTAL REFERENCES

1. TCGA. Comprehensive genomic characterization defines human glioblastoma genes and core pathways. Nature. 2008; 455: 1061-1068.
2. Taylor BS, Schultz N, Hieronymus H, Gopalan A, Xiao Y, Carver BS, Arora VK, Kaushik P, Cerami E, Reva B, Antipin Y, Mitsiades N, Landers T, Dolgalev I, Major JE, Wilson M, et al. Integrative genomic profiling of human prostate cancer. Cancer cell. 2010; 18: 11-22.
3. Hieronymus, H., Schultz, N., Gopalan, A., Carver, B.S., Chang, M.T., Xiao, Y., Heguy, A., Huberman, K., Bernstein, M., Assel, M. 2014. Copy number alteration burden predicts prostate cancer relapse. Proceedings of the National Academy of Sciences 111:11139-11144.
4. Zhu, J., Zhang, B., Smith, E., Drees, B., Brem, R., Kruglyak, L., Bumgarner, R., Schadt, E. 2008. Integrating large-scale functional genomic data to dissect the complexity of yeast regulatory networks. Nature genetics 40:854-861.
5. Zhu, J., Lum, P., Lamb, J., GuhaThakurta, D., Edwards, S., Thieringer, R., Berger, J., Wu, M., Thompson, J., Sachs, A., et al. 2004. An integrative genomics approach to the reconstruction of gene networks in segregating populations. Cytogenetic and genome research 105:363-374.
6. Zhu J, Wiener MC, Zhang C, Fridman A, Minch E, Lum PY, Sachs JR and Schadt EE. Increasing the power to detect causal associations by combining genotypic and expression data in segregating populations. PLoS Comput Biol. 2007; 3: e69.
7. Zhu J, Sova P, Xu Q, Dombek KM, Xu EY, Vu H, Tu Z, Brem RB, Bumgarner RE and Schadt EE. Stitching together multiple data dimensions reveals interacting metabolomic and transcriptomic networks that modulate cell regulation. PLoS Biol. 2012; 10: e1001301.
8. Pearl J. (1988). Probabilistic reasoning in intelligent systems : networks of plausible inference. (San Mateo, Calif.: Morgan Kaufmann Publishers).
9. Madigan, D.a.Y., J. 1995. Bayesian graphical models for discrete data. International Statistical Review 63:215-232.
10. Schwarz, G. 1978. Estimating the dimension of a model. Annals of Statistics 6:461-464.
11. Zhu, J., Wiener, M.C., Zhang, C., Fridman, A., Minch, E., Lum, P.Y., Sachs, J.R., Schadt, E.E. 2007. Increasing the Power to Detect Causal Associations by Combining Genotypic and Expression Data in Segregating Populations. PLoS Comput Biol 3:e69.
12. Peri S, Navarro JD, Kristiansen TZ, Amanchy R, Surendranath V, Muthusamy B, Gandhi TK, Chandrika KN, Deshpande N, Suresh S, Rashmi BP, Shanker K, Padma N, Niranjan V, Harsha HC, Talreja N, et al. Human protein reference database as a discovery resource for proteomics. Nucleic Acids Res. 2004; 32(Database issue):D497-501.
13. Franceschini A, Szklarczyk D, Frankild S, Kuhn M, Simonovic M, Roth A, Lin J, Minguez P, Bork P, von Mering C and Jensen LJ. STRING v9.1: protein-protein interaction networks, with increased coverage and integration. Nucleic Acids Res. 2013; 41(Database issue):D808-815.
14. Lee I, Blom UM, Wang PI, Shim JE and Marcotte EM. Prioritizing candidate disease genes by network-based boosting of genome-wide association data. Genome research. 2011; 21: 1109-1121.
15. Kanehisa M and Goto S. KEGG: kyoto encyclopedia of genes and genomes. Nucleic Acids Res. 2000; 28 :27-30.
16. Subramanian, A., Tamayo, P., Mootha, V.K., Mukherjee, S., Ebert, B.L., Gillette, M.A., Paulovich, A., Pomeroy, S.L., Golub, T.R., Lander, E.S., et al. 2005. Gene set enrichment analysis: a knowledge-based approach for interpreting genome-wide expression profiles. Proceedings of the National Academy of Sciences of the United States of America 102:15545-15550.
17. Ashburner M, Ball CA, Blake JA, Botstein D, Butler H, Cherry JM, Davis AP, Dolinski K, Dwight SS, Eppig JT, Harris MA, Hill DP, Issel-Tarver L, Kasarskis A, Lewis S,

Matese JC, et al. Gene ontology: tool for the unification of biology. The Gene Ontology Consortium. Nat Genet. 2000; 25: 25-29

18. Futreal PA, Coin L, Marshall M, Down T, Hubbard T, Wooster R, Rahman N and Stratton MR. A census of human cancer genes. Nature reviews Cancer. 2004; 4: 177-183

19. Setlur, S.R., Mertz, K.D., Hoshida, Y., Demichelis, F., Lupien, M., Perner, S., Sboner, A., Pawitan, Y., Andrén, O., Johnson, L.A., et al. 2008. Estrogen-dependent signaling in a molecularly distinct subclass of aggressive prostate cancer. Journal of the National Cancer Institute 100:815-825.

20. Hieronymus H, Lamb J, Ross KN, Peng XP, Clement C, Rodina A, Nieto M, Du J, Stegmaier K, Raj SM, Maloney KN, Clardy J, Hahn WC, Chiosis G and Golub TR. Gene expression signature-based chemical genomic prediction identifies a novel class of HSP90 pathway modulators. Cancer cell. 2006; 10: 321-330.

SUPPLEMENTARY FIGURES AND TABLES

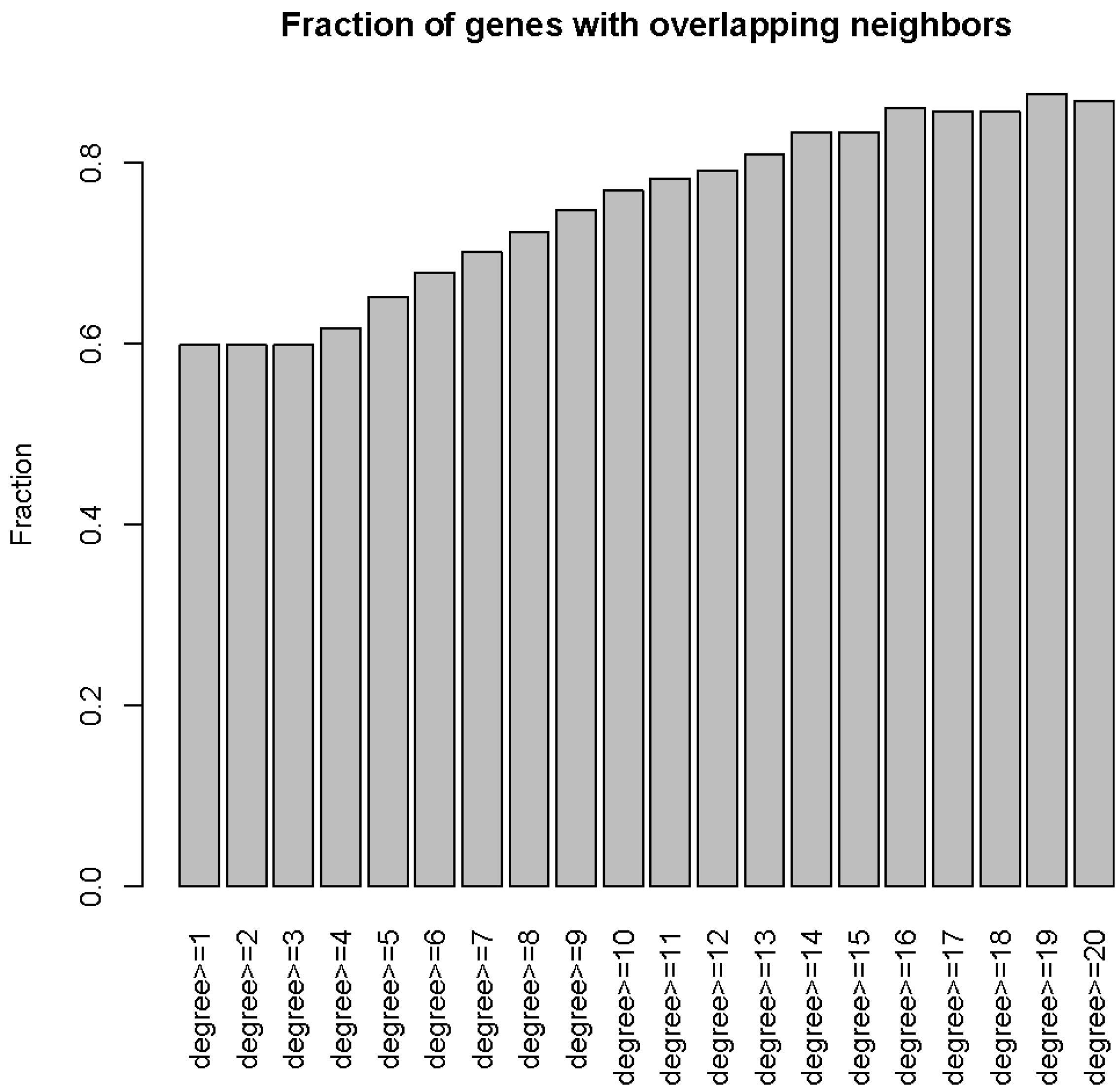

Supplementary Figure S1: Fraction of genes with similar network neighborhood in TCGA and Taylor's datasets as grouped by their node degrees.

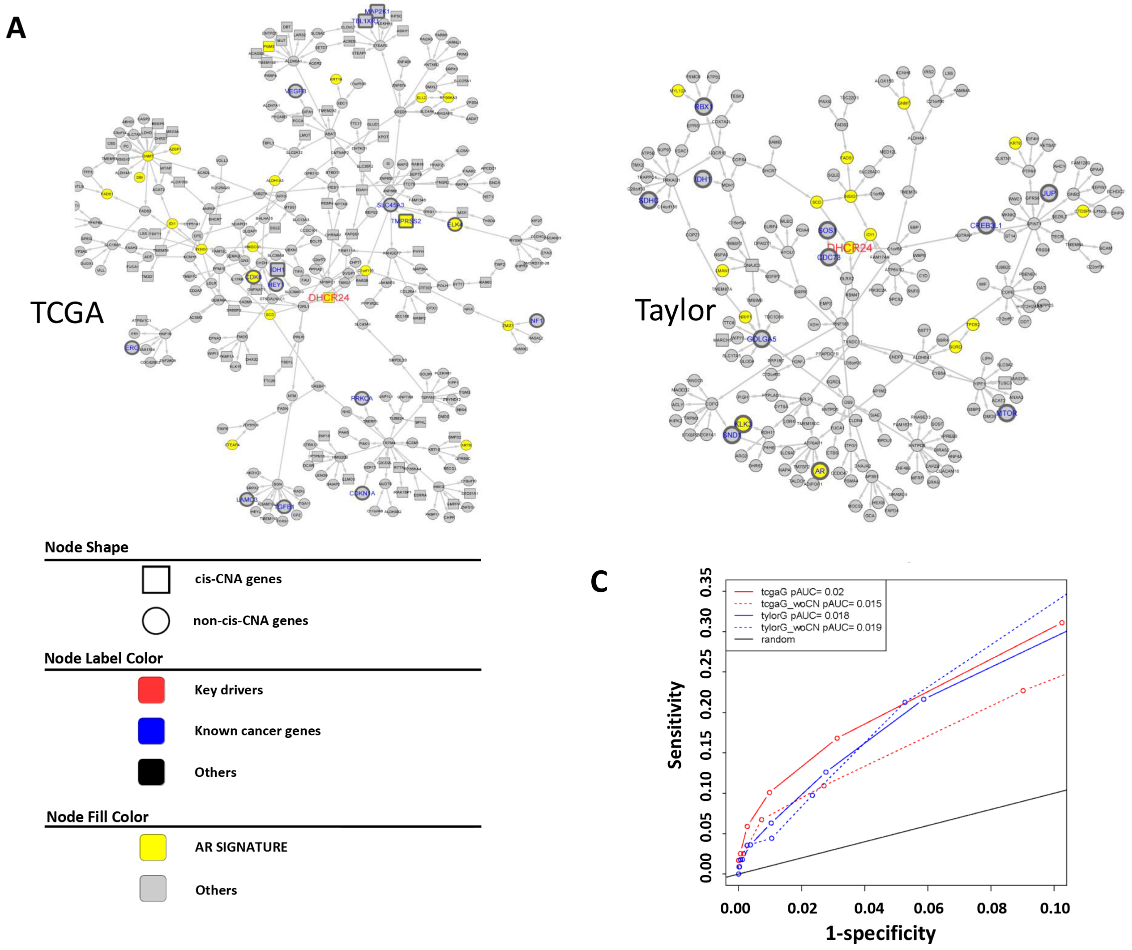

Supplementary Figure S2: A and **B.** DHCR24 subnetwork extracted from TCGA IMBN (A) and Tylor IMBN (B). Nodes of yellow color represent the known AR signature genes. Genes known to be cancer related are labeled in larger font size. Square nodes represent genes regulated by cis CNA. **C.** Evaluation of DHCR24 subnetworks using AR signature genes through ROC like curve and partial AUC.

A

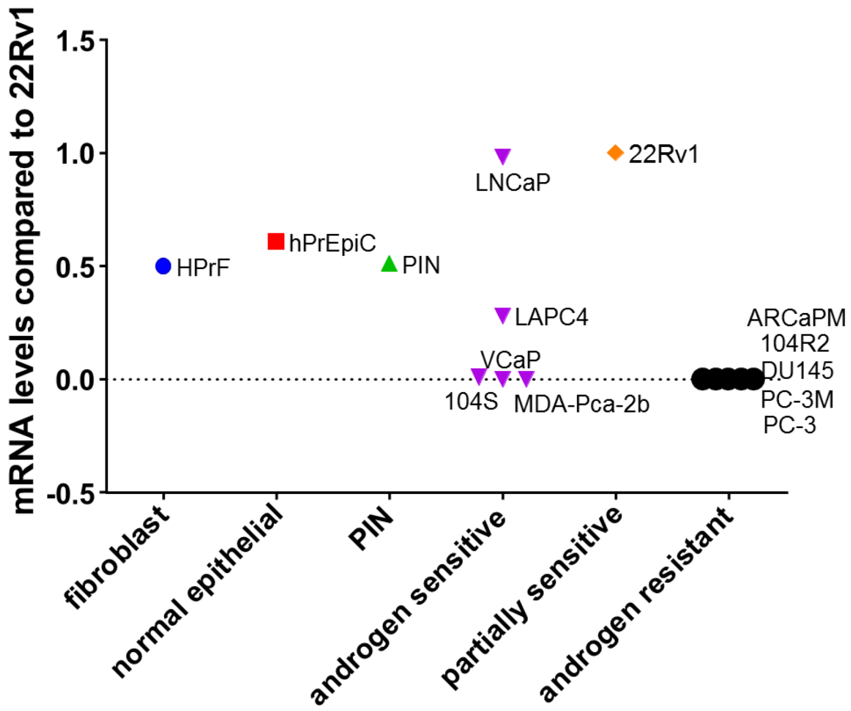

Supplementary Figure S3: Expression of *NLGN4Y* transcripts in a panel of PCa cell lines. Cellular mRNA expression was examined by qRT-PCR and the expression level was normalized to *NLGN4Y* level in 22Rv1 cells. *NLGN4Y* transcripts were not detected in 8 cell lines as shown above.

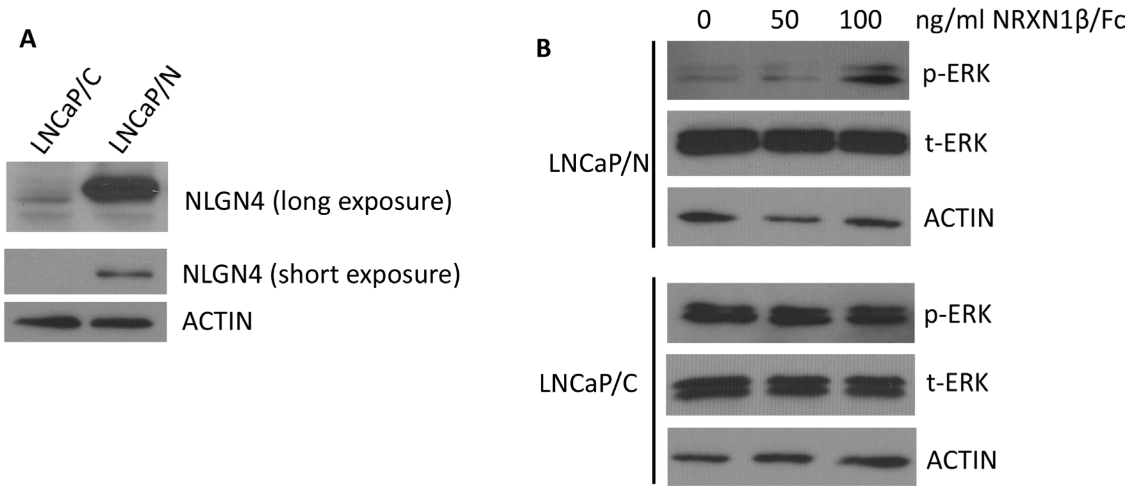

Supplementary Figure S4: A. Short and long exposure results of western blot analysis of expression of exogenous NLGN4Y in LNCaP cells. **B.** Treatment of LNCaP/N cells with Neurexin 1β/Fc decoy receptor induced phosphorylation of ERK.

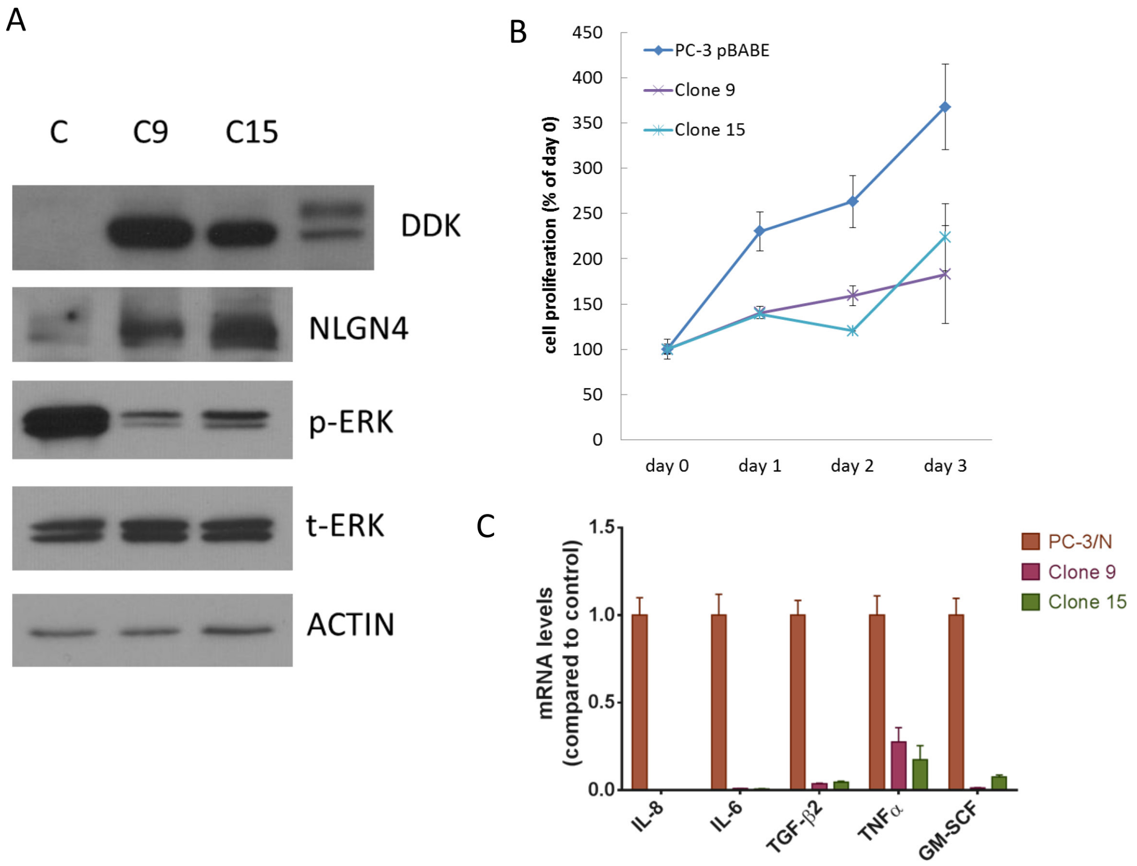

Supplementary Figure S5: A. Monoclonal PC3/N cells showed dramatically decreased ERK phosphorylation. **B.** Monoclonal PC3/N cells proliferated slower. **C.** Expression of several pro-inflammatory cytokines was suppressed in monoclonal PC-3/N cells.

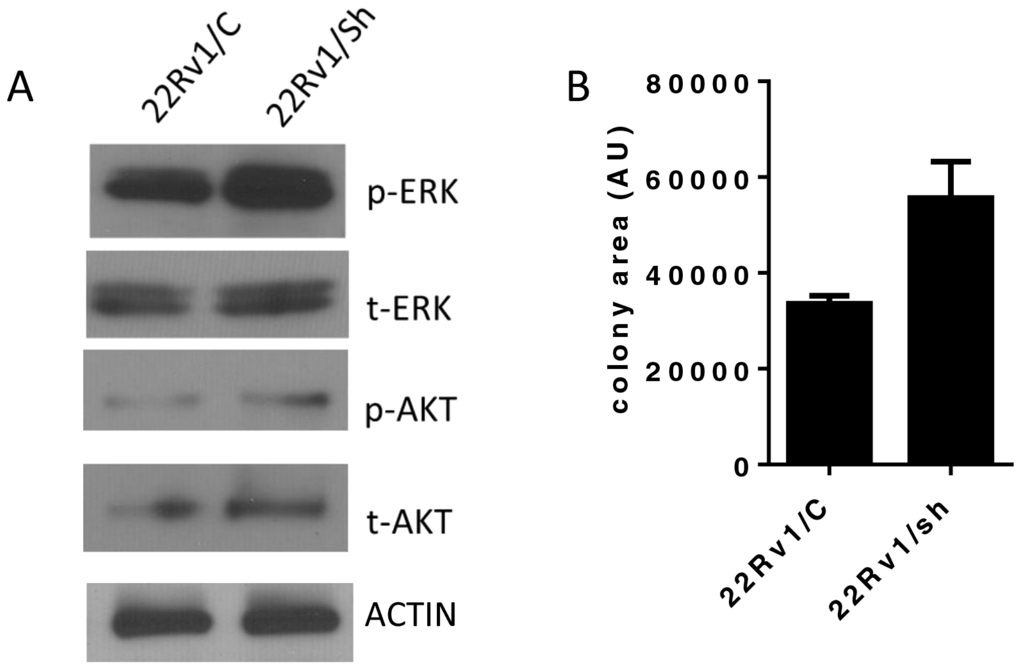

Supplementary Figure S6: NLGN4Y regulated ERK and cell proliferation in 22Rv1 cells. A. Knockdown of NLGN4Y mRNA in 22Rv1 cells increased ERK phosphorylation. **B.** Knockdown of NLGN4Y mRNA in 22Rv1 cells increased cell proliferation.

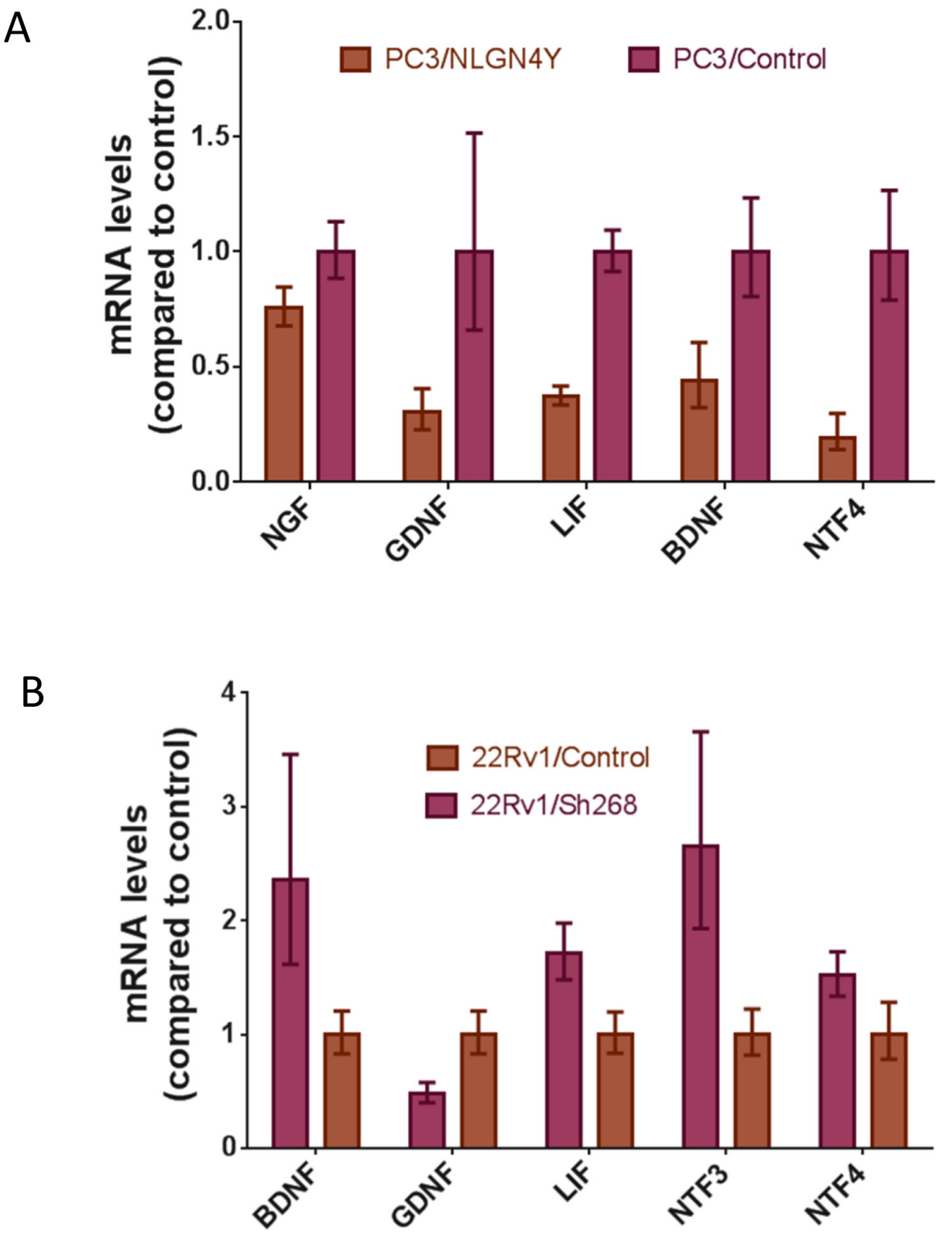

Supplementary Figure S7: NLGN4Y expression decreased the mRNA levels of several neurotropic factors in PC-3 cells. A. and consistently, NLGN4Y shRNA upregulated the expression of these genes **B.**

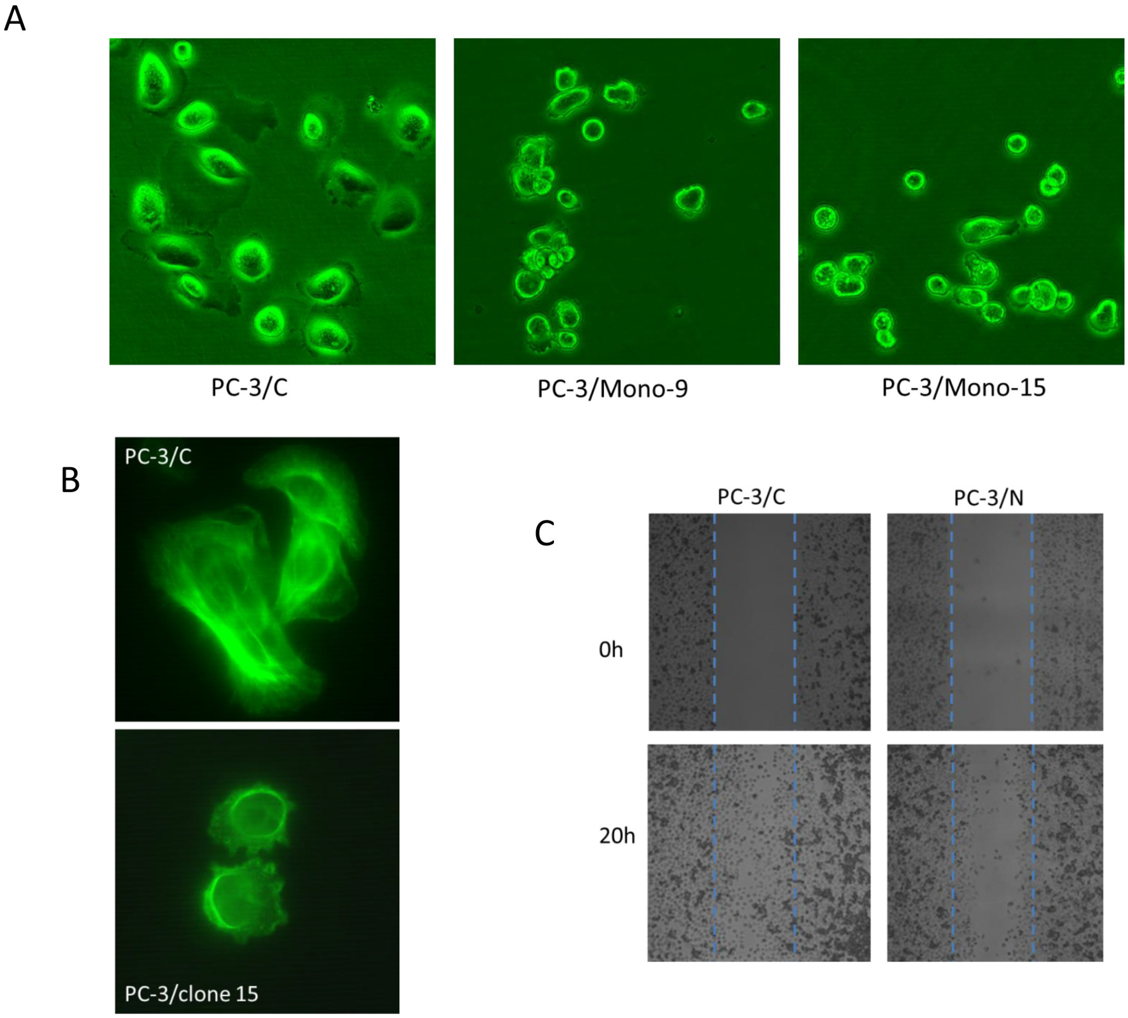

Supplementary Figure S8: A. Defects in cell spreading in monoclonal PC-3/N cells (clone 9 and 15). **B.** Defects in F-actin organization in monoclonal PC-3/N cells (clone 15). **C.** Defects in cell migration in monoclonal PC-3/N cells (clone 15) as shown by a wound healing assay.

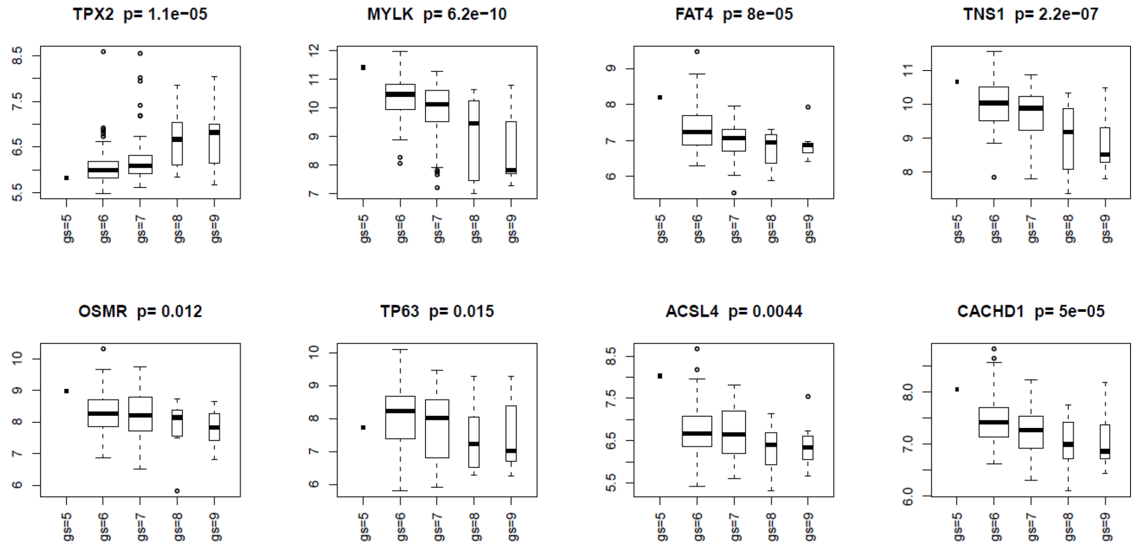

Supplementary Figure S9: Barplot of gene expression of key regulators for samples with different Gleason scores. P-value was calculated based on the F test for the association between gene expression and Gleason score.

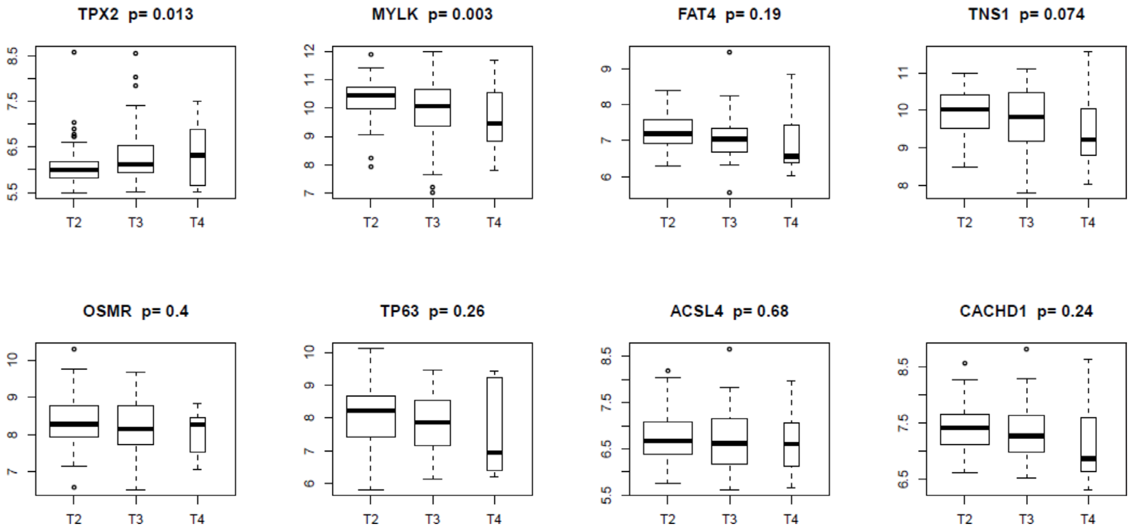

Supplementary Figure S10: Barplot of gene expression of key regulators for samples with different tumor stages. P-value was calculated based on the F test for the association between gene expression and tumor stage.

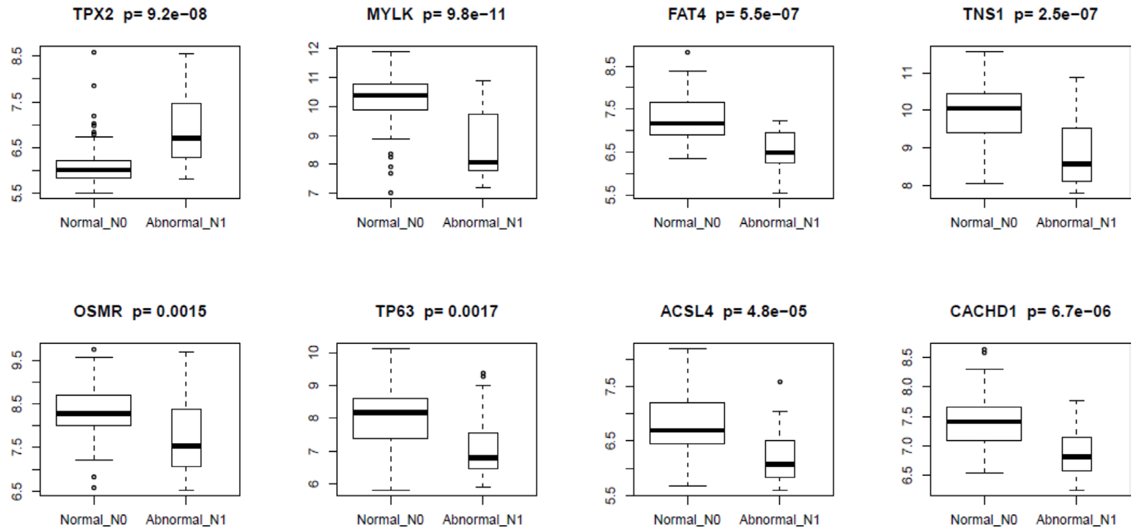

Supplementary Figure S11: Barplot of gene expression of key regulators for samples with different lymph node status. P-value was calculated based on the F test for the association between gene expression and lymph node status.

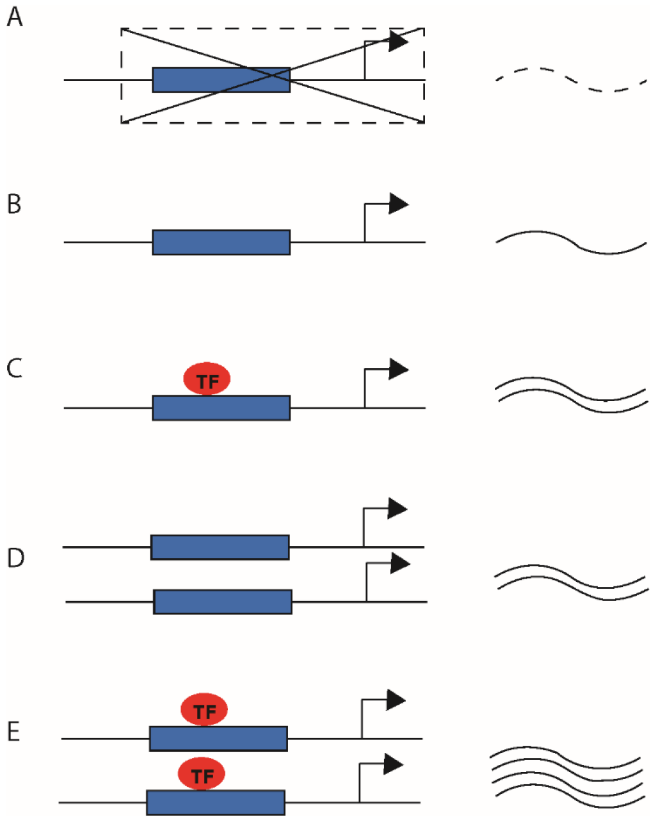

Supplementary Figure S12: Gene expression variation of a gene is caused by CNA of the gene and binding of transcription factors (TFs) to its promoter region. **A.** low gene expression due to a low copy number of the gene; **B.** low gene expression due to none TF binding; **C.** high gene expression due to TF binding at the promoter region; **D.** high gene expression due to gene copy number amplification; **E.** higher gene expression due to gene copy number amplification with TF binding at the promoter region.

Supplementary Table S1: The estimated accuracy of PCa IMBNs using different benchmark datasets

Reference databases		TCGA dataset		Taylor dataset	
		expression & CNA	expression only	expression & CNA	expression only
Gene network	HPRD [81]	1.2% (0.1%±0.05%)	1.1% (0.11%±0.05%)	1% (0.11%±0.06%)	1.2% (0.12%±0.07%)
	HumanNet [24]	7.5% (0.61%±0.07%)	7% (0.59%±0.06%)	7.3% (0.63%±0.1%)	7% (0.64%±0.1%)
	STRING [22]	7% (0.29%±0.07%)	6.5% (0.3%±0.05%)	6.1% (0.33%±0.09%)	6.3% (0.34%±0.09%)
Gene sets	KEGG [25]	26% (5.1%±0.6%)	24% (4.8%±0.6%)	32% (4.9%±0.6%)	30% (4.8%±0.8%)
	MsigDB Hallmark [26]	34% (7.5%±0.6%)	32% (7.2%±0.6%)	28% (7.4%±1%)	28% (7.3%±0.9%)
	GO* [27]	14% (4.3%±0.4%)	13% (4.4%±0.4%)	12% (4.3%±0.5%)	12% (4.2%±0.5%)

*GO gene sets with size >=200 are excluded.

Numbers in the parentheses are the mean and standard deviation of accuracies of random networks generated by permuting gene names in the corresponding IMBN.

Supplementary Table S2: A list of "high confidence" prostate cancer genes defined as those included in more than one of the prostate cancer related gene sets listed in Supplementary Table S3

See Supplementary File 1

Supplementary Table S3: Gene sets in MsigDB used to generate a list of high confident prostate cancer genes

Geneset Name	GeneNum
CHANDRAN_METASTASIS_DN	306
CHANDRAN_METASTASIS_UP	221
KEGG_PROSTATE_CANCER	89
LI_PROSTATE_CANCER_EPIGENETIC	30
LIU_PROSTATE_CANCER_DN	482
LIU_PROSTATE_CANCER_UP	96
TOMLINS_METASTASIS_DN	20
TOMLINS_METASTASIS_UP	14
TOMLINS_PROSTATE_CANCER_DN	40
TOMLINS_PROSTATE_CANCER_UP	40
WALLACE_PROSTATE_CANCER_DN	6
WALLACE_PROSTATE_CANCER_UP	20
WANG_HCP_PROSTATE_CANCER	111
YEGNASUBRAMANIAN_PROSTATE_CANCER	128

The high confident prostate cancer genes are defined as those included in more than one of the above gene sets.

Supplementary Table S4: Canonical pathways enriched in ERG subnetworks (MsigDB)

	TCGA	Tylor
REACTOME_NEURONAL_SYSTEM (158)	6.E-03	1.E-02
REACTOME_TRNA_AMINOACYLATION (26)	1.E+00	2.E-04
REACTOME_CYTOSOLIC_TRNA_AMINOACYLATION (17)	1.E+00	2.E-03
KEGG_PURINE_METABOLISM (113)	4.E-01	5.E-03
REACTOME_NITRIC_OXIDE_STIMULATES_GUANYLATE_CYCLASE (21)	1.E+00	2.E-03
KEGG_AMINOACYL_TRNA_BIOSYNTHESIS (24)	1.E+00	3.E-03
MIPS_MULTISYNTHETASE_COMPLEX (8)	1.E+00	3.E-03
REACTOME_PLATELET_HOMEOSTASIS (56)	1.E+00	3.E-03
PID_AR_TF_PATHWAY (35)	4.E-01	1.E-02
MIPS_P2X7_RECEPTOR_SIGNALLING_COMPLEX (9)	5.E-03	1.E+00

Note: Numbers in each cell represent p-values form fisher's exact test. TCGA: ERG subnetwork extracted from TCGA IMBN. Tylor: ERG subnetwork extracted from Tylor IMBN.

Supplementary Table S5: Hallmark pathways enriched in BCR subnetworks (MsigDB)

	tcga_pos	tcga_neg	tylor_pos	tylor_neg	combined_ pos	combined_ neg
HALLMARK_E2F_ TARGETS (146)	1.E-54	1.E+00	2.E-14	1.E+00	1.E-52	1.E+00
HALLMARK_G2M_ CHECKPOINT (142)	7.E-43	1.E+00	2.E-19	1.E+00	2.E-44	1.E+00
HALLMARK_TNFA_ SIGNALING_VIA_NFKB (169)	1.E+00	2.E-12	7.E-01	4.E-16	1.E+00	4.E-19
HALLMARK_MITOTIC_ SPINDLE (156)	2.E-13	1.E+00	2.E-09	9.E-01	7.E-15	1.E+00
HALLMARK_ EPITHELIAL_ MESENCHYMAL_ TRANSITION (179)	9.E-01	2.E-03	9.E-01	2.E-11	7.E-01	4.E-11
HALLMARK_ MYOGENESIS (159)	9.E-01	7.E-11	1.E+00	3.E-05	9.E-01	6.E-09
HALLMARK_HYPOXIA (173)	1.E+00	2.E-05	1.E+00	5.E-04	1.E+00	5.E-07
HALLMARK_APOPTOSIS (130)	8.E-01	6.E-03	8.E-01	2.E-06	8.E-01	4.E-06
HALLMARK_UV_ RESPONSE_DN (124)	9.E-01	2.E-02	9.E-01	2.E-04	8.E-01	9.E-07
HALLMARK_ SPERMATOGENESIS (78)	3.E-06	1.E+00	2.E-02	9.E-01	5.E-05	8.E-01
HALLMARK_ESTROGEN_ RESPONSE_EARLY (174)	1.E+00	3.E-02	1.E+00	8.E-04	1.E+00	8.E-05
HALLMARK_ COAGULATION (101)	1.E+00	5.E-02	1.E+00	3.E-04	1.E+00	1.E-04
HALLMARK_MYC_ TARGETS_V1 (143)	2.E-05	1.E+00	5.E-01	1.E+00	1.E-03	1.E+00
HALLMARK_IL6_JAK_ STAT3_SIGNALING (62)	1.E+00	3.E-02	1.E+00	4.E-03	1.E+00	5.E-03
HALLMARK_KRAS_ SIGNALING_UP (169)	1.E+00	4.E-02	7.E-01	4.E-02	1.E+00	1.E-03
HALLMARK_IL2_STAT5_ SIGNALING (168)	1.E+00	9.E-03	9.E-01	1.E-01	1.E+00	5.E-03
HALLMARK_ INFLAMMATORY_ RESPONSE (154)	1.E+00	8.E-02	1.E+00	6.E-02	1.E+00	9.E-03
HALLMARK_ INTERFERON_GAMMA_ RESPONSE (170)	1.E+00	1.E+00	1.E+00	9.E-03	1.E+00	4.E-02

Note: Numbers in each cell represent p-values form fisher's exact test. tcga_pos/tylor_pos/combined_pos: subnetworks positively associated with BCR extracted from TCGA IMBN/Tylor IMBN/combined IMBN. tcga_neg/tylor_neg/combined_neg: subnetworks negatively associated with BCR extracted from TCGA IMBN/Tylor IMBN/combined IMBN.

Supplementary Table S6: A list of 813 known cancer genes (not restricted to prostate cancer) collected from cancer gene census [18] and KEGG cancer pathways [15]

See Supplementary File 2

Supplementary Table S7: A list of 87 TMPRSS2-ERG fusion signature genes (comparing PCa patients with and without the fusion) collected from a previous study [19]

See Supplementary File 3

Supplementary Table S8: A list of 157 AR signature genes collected from multiple sources [16, 20]

See Supplementary File 4
